# Transcriptomic Analysis and Comparative Analysis of Gene Families Related to Environmental Adaptation in Two Grylloblattodea Species: *Galloisiana sinensis* and *Grylloprimevala jilina*

**DOI:** 10.1002/ece3.72260

**Published:** 2025-10-07

**Authors:** Yanhan Zhou, Kaipeng Zhang, Luyao Yu, Yuxin Zhou, Yuetong Xiao, Lin Zhou, Xiaoyan Zhu, Taoqi Wang, Qi Chen, Bingzhong Ren

**Affiliations:** ^1^ Jilin Provincial Key Laboratory of Animal Resource Conservation and Utilization, School of Life Sciences Northeast Normal University Changchun China; ^2^ Key Laboratory of Vegetation Ecology, Ministry of Education Northeast Normal University Changchun China; ^3^ Jilin Provincial Engineering Laboratory of Avian Ecology and Conservation Genetics Northeast Normal University Changchun China; ^4^ Research Institute of Forest Protection Jilin Provincial Academy of Forestry Sciences Changchun China; ^5^ Institute of Plant Protection Jilin Academy of Agricultural Science/Jilin Key Laboratory of Agricultural Microbiology/Key Laboratory of Integrated Pest Management on Crops in Northeast China Ministry of Agriculture and Rural Areas Gongzhuling China

**Keywords:** environmental adaptation, *Galloisiana sinensis*, Grylloblattodea, *Grylloprimevala jilina*, transcriptome, wing regression

## Abstract

Notoptera, as a unique ancient insect lineage, elucidates the linkage between insect biodiversity and geological history. Members of this order are recognized as National First‐Class Protected Animals in China. Comparing environmental adaptation (traits evolved for survival/reproduction in a specific environment) genes in *Galloisiana sinensis* and *Grylloprimevala jilina*, closely related species with distinct habitats, elucidates Notoptera evolution. Our study identified chemosensory genes, temperature adaptation‐related genes, vision‐related genes, and winged morph differentiation‐related genes in *G. sinensis* and compared them with those in *G. jilina*. Both species exhibit gene loss, possibly owing to winglessness. *G. sinensis* has fewer odorant receptors, odorant‐binding proteins, and chemosensory proteins but more gustatory receptors, ionotropic receptors, and sensory neuron membrane proteins, reflecting habitat‐specific adaptations. Compared with *G. jilina*, *G. sinensis* also exhibits more vision‐related genes and an enhanced photosensitive system. *G. sinensis* has fewer heat shock proteins and transient receptor potential proteins but more DnaJ molecular chaperones (DnaJ proteins), suggesting improved molecular chaperone function for broader temperature tolerance. Despite wing loss, *Wnt6*, *Wnt7b*, and *Wnt16* gene remnants remain, with *G. sinensis* having fewer of these, indicating advanced wing regression. This work reveals the genetic basis of adaptation and evolution, providing key insights into Notoptera's evolutionary trajectory.

## Graphical abstract

Notoptera, a singular ancient insect lineage, is vital for exploring insect environmental adaptation, evolution, and its geological history link, recognized as a national first‐class protected animal. Comparing environmental adaptation genes in *Galloisiana sinensis* and *Grylloprimevala jilina*, closely related yet habitat‐disparate species, illuminates Notoptera's evolution.

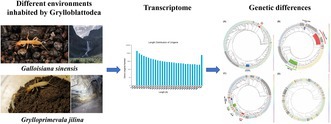

## 1 Introduction

**FIGURE 1:**
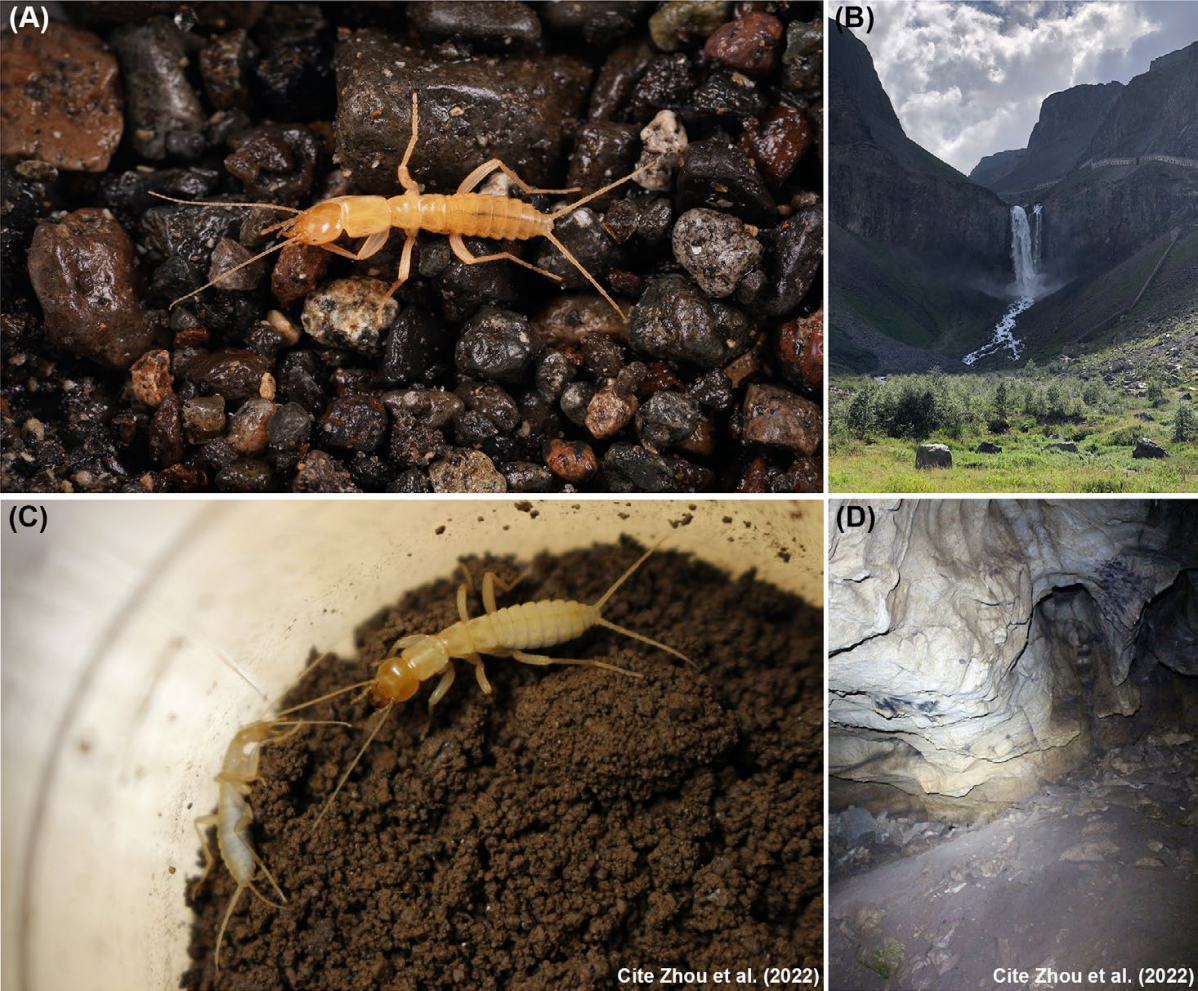
Photographs of the biology and habitat of *Galloisiana sinensis* and *Grylloprimevala jilina*. (A) Photograph of the biology of *G. sinensis* . (B) Photograph of the habitat of *G. sinensis* on a rock pile of the landslide on the left side of the Tianchi waterfall, Changbai Mountain. (C) and (D) are credited to previous studies, featuring a photograph of the biology of *G. jilina* and a photograph of its cave habitat (Zhou, Chen, et al. 2023).

As the sole extant relictual group (a taxon that represents the surviving remnant of an ancient lineage) in Insecta, Notoptera exhibits the lowest diversity among insect groups. It currently comprises 40 taxa (species and subspecies) in six genera, including only four species recorded in China (Zhou, Zhou, et al. 2023). Grylloblattidae represent an ancient insect lineage with unique biological characteristics. These insects are distributed primarily in high‐altitude or high‐latitude cold regions of the Northern Hemisphere and exhibit remarkable cold adaptation traits. Their life history features include an extended developmental cycle, a narrow thermal activity range (optimal temperature of 1°C–4°C), and distinctive seasonal activity patterns (Jarvis et al. 2004). Therefore, the order Grylloblattodea is regarded as an order of “living fossils” (species that combine both phenotypic stasis and phylogenetic relict status) (Casane and Laurenti 2013) among contemporary insects and is acknowledged as “one of the most significant extant insect groups” (Walker 1937; Bai et al. 2010), which has led to its classification as Category I protected species under China's National List of Key Protected Wildlife (Zhou, Zhou, et al. 2023). Published evolutionary studies comparing fossil families (extinct taxonomic families known exclusively from fossilized remains) (Tihelka et al. 2021) with extant ones (taxonomic families that currently have living representatives) (Condamine et al. 2016) have demonstrated that Notoptera thrived predominantly during the Permian and Triassic periods (Eberhard et al. 2018), undergoing an evolutionary journey characterized by a decrease in body size, a transition from winged to wingless forms, and a shift from widespread to restricted distributions, all while exhibiting high sensitivity to environmental factors such as temperature and humidity (Bai et al. 2010). As a result, Notoptera species serve as key models for studying evolutionary origins, adaptation mechanisms, and insect–geological relationships and are thereby classified as a threatened group on the verge of extinction (Bai et al. 2010). *Galloisiana sinensis* , the focal organism of this study (Figure 1A), classified within the order Notoptera and family Grylloblattodea, was discovered in landslide rubble beneath the precipitous cliffs near the Tianchi Waterfall, sitting at the apex of the Changbai Mountain range. This area has exceptionally cold annual temperatures, ranging from −29.9°C to −29.6°C (Bai et al. 2010; Zhang et al. 2021). *Grylloprimevala jilina* (Notoptera: Grylloblattodea), the focal organism of this study (Figure 1C), is a true troglobitic insect (a species that is an obligate cave dweller) exhibiting characteristic adaptations lacking body pigmentation, possessing degenerated compound eyes and ocelli, exhibiting enhanced body sensors, and showing a marked preference for high‐humidity/low‐temperature environments (Zhou, Chen, et al. 2023). The caves maintain a constant temperature of 16°C throughout the year. Despite their vastly different living conditions, these insects are closely related phylogenetically (Zhou, Chen, et al. 2023); thus, comparative studies of these insects are essential for elucidating the evolutionary trajectory of the order Grylloblattodea.

Environmental selective pressures are crucial drivers of insect evolution (Hoffmann and Hercus 2000). These pressures are mediated through chemosensory genes that detect chemical cues, temperature adaptation‐related genes that respond to thermal fluctuations, and vision‐related genes that perceive light changes. Additionally, wing morph differentiation‐related genes enable adaptive responses to habitat variability. These genetic systems allow insects to directly perceive and rapidly adapt to selective environmental forces. For example, in *Anopheles gambiae* , the odorant receptor OR4 has undergone positive selection to increase sensitivity to human‐derived volatiles, driving its specialization in human hosts (McBride et al. 2014). In *Drosophila melanogaster* , allelic variation in HSP70 correlates with latitudinal clines—populations in tropical regions retain heat‐inducible HSP70 variants, whereas temperate strains evolve constitutively expressed isoforms for rapid cold hardening (Sørensen et al. 2007). *Ptomaphagus hirtus* shows degenerate Opsin genes due to relaxed selection in the dark (Friedrich et al. 2011).

Among chemosensory receptors, odorant receptors (ORs), an essential class of chemoreceptors, mediate pheromone and volatile chemical detection. They transmit signals into olfactory glomeruli upon recognition of odorant molecules transported by odorant‐binding proteins (OBPs) (Leal 2013; Ribeiro et al. 2014). Chemosensory proteins (CSPs) function like OBPs and can also exhibit binding functions (Wicher and Miazzi 2021). Gustatory receptors (GRs), which are expressed mainly in taste organs and serve as receptors for chemical substances in food, are protein families that include the fructose taste receptor family, CO_2_ family, bitterness family, and sugar receptor family (Miyamoto and Amrein 2014; Xu et al. 2017). Ionotropic receptors (IRs) participate in olfaction or taste sensing and are also sensitive to humidity (Benton et al. 2009; Croset et al. 2010). Sensory neuron membrane protein (SNMP) aids in the OR‐mediated perception of pheromones (Benton et al. 2007). Among vision‐related genes, visual system homeobox (VSX) is crucial for the differentiation and function of photoreceptor cells in the retina (Joly et al. 2016). Opsins are the key molecules in the visual process that convert light signals into neural signals (Huang et al. 2024). Phosphodiesterase (PDE6D) plays a significant role in modulating the sensitivity of retinal receptor cells to light (Link and Collery 2015). Retinol binding protein (RBP) ensures a sufficient supply of retinol to the retina, supporting the regeneration of visual pigments and the maintenance of visual sensitivity (Pelosi et al. 2018). Among temperature adaptation‐related genes, trehalose transporter (Tret) genes facilitate the transmembrane transport of trehalose, which serves not only as a form of energy storage but also as an osmoprotectant (Jain and Kumar 2010). The expression and function of Tret genes are crucial for maintaining the osmotic balance between cells and their environment, especially under environmental stresses such as drought, high temperature, or freezing (Zhou et al. 2022). Heat shock proteins (HSPs) are a class of proteins abundantly produced when organisms encounter stressors such as high temperature, oxidative stress, or infection. Their primary roles include ensuring the stability and correct folding of intracellular proteins and participating in the repair or degradation of damaged proteins (Dong et al. 2022), whereas DNAj acts as a cochaperone to HSPs (Yang et al. 2022). Transient receptor potential (TRP) channels are responsible for sensing and responding to a variety of physical stimuli (e.g., temperature and mechanical forces) and chemical cues (e.g., pH changes and osmotic pressure fluctuations) (Wei et al. 2015; Diver et al. 2022). Among the wing morph differentiation‐related genes, Wingless (Wnt) genes are intimately tied to wing development and the adult eclosion process (Swarup and Verheyen 2012). In this study, we identified these environmentally adaptive genes in *G. sinensis* and compared them with those of *G. jilina*, providing key data for elucidating the evolutionary process of the order Grylloblattodea.

## 2 Materials and Methods

### 2.1 Insects

*Galloisiana sinensis* was gathered from the landslide rock pile on the left side of the Tianchi waterfall, Changbai Mountain (42°2′29.8″ N, 128°3′38.5″ E, elevation 1942.3 m, 2023‐VII‐21). In Ji'an city, Jilin Province, China, a primeval forest containing a natural cave where *G. jilina* was found in a darkened part inside a pile of rocks (Figure 1; Zhou, Chen, et al. 2023).

### 2.2 RNA‐Seq and Library Construction

The *G. sinensis* transcriptome was analyzed. The samples were prepared from four tissues of *G. sinensis* (heads, thoraxes, abdomens, and legs); owing to the classification of the species as Category I nationally protected animal, sample collection is extremely restricted, making it impossible to quantify expression in the antennae or cercis separately. The samples were then frozen in liquid nitrogen and stored at −80°C until RNA extraction. Each tissue had three biological replicates. TRIzol reagent (Invitrogen, Carlsbad, CA, USA) was used to extract total RNA from tissue samples in accordance with the manufacturer's instructions. The integrity of the RNA was assessed using a NanoDrop 2000 (Thermo Fisher Scientific, Waltham, MA, USA) and 1% agarose gel electrophoresis (Zhou, Zhou, et al. 2023). Beijing Baimaike Biotechnology Co. Ltd., China, used the Illumina NovaSeq platform, a second‐generation high‐throughput sequencing technology, to perform transcriptome sequencing of four distinct *G. sinensis* tissues. In accordance with the manufacturer's instructions, sequencing libraries were created using the NEBNext Ultra RNA Library Prep Kit for Illumina (NEB, USA), and index codes were added to each sample to identify its sequence. Following the sequencing of the libraries on an Illumina HiSeq 2000 PE150 platform, 150 bp paired‐end reads were produced. Finally, the raw reads were acquired.

### 2.3 Assembly of the Transcriptome and Annotation of Functional Genes

To acquire sequence clean reads, low‐quality and junction sequences were eliminated. For transcriptome assembly, Trinity (r20131110)'s left.fq and right.fq functions were used with all of their default settings. All of the sample total reads were combined into a single transcriptome. The transcripts were clustered using the Trinity_cluster_with_id_com Pl. The longest isoform from each Trinity assembly was classified as a unigene (Grabherr et al. 2011).

Total reads were used to produce the final unigene dataset. The quality of the generated transcriptome sequences was assessed using BUSCO v. 3.0.2 (Simão et al. 2015). The generated unigenes were compared using the DIAMOND (Buchfink et al. 2015) software using the NCBI nonredundant (NR) database (Deng et al. 2006). The functional labeling of the unigenes was performed on the basis of previous experience (Zhou, Zhou, et al. 2023).

### 2.4 Identification of Genes Involved in Adaptation to the Environment

Preliminary screening was conducted on *G. sinensis* chemosensory genes, vision‐related genes, and temperature adaptation‐related genes on the basis of the annotation results for the transcriptome database. Our screening method for these genes was based on the screening method previously used for *G. jilina* (Zhou, Zhou, et al. 2023). The following chemosensory genes have been identified: GRs, IRs, OBPs, CSPs, and SNMPs (Sánchez‐Gracia et al. 2009; Leal 2013). Among the genes connected to vision, we identified VSX (Sanes and Zipursky 2010). We found the following genes to be associated with temperature adaptation: TRPs, HSPs, DnaJs, and Trets (Tang et al. 2018; Chen et al. 2021; Liu and Zhang 2022). Wnts were ultimately identified as genes linked to the differentiation of wing morphology. The transcriptome database annotation results were merged with manual annotations by the NCBI‐blast‐2.11.0 basic local comparison search tool to uncover candidate genes linked to temperature adaptation and vision and *G. sinensis* chemosensory genes. The ORF finder tool (https://www.ncbi.nlm.nih.gov/orffinder/) was used to estimate the relevant open reading frames (ORFs) and amino acid sequences of gene transcripts on the basis of the final annotation findings. The sequences were manually rechecked against the GenBank nonredundant (NR) protein database using the BLASTx program (*E* value < 10^−5^) after being uploaded to NCBI BLAST (https://blast.ncbi.nlm.nih.gov/Blast.cgi) (Zhou, Han, et al. 2023).

### 2.5 Phylogenetic Analysis

We selected phylogenetically related species and representative model insects to construct a phylogenetic tree: *Acyrthosiphon pisum* , *Anopheles gambiae* , *Blattella germanica* , *Bombyx mori* , *Culex pipiens* , *Cryptotermes secundus*, *Drosophila melanogaster* , *Drosophila pseudoobscura* , *Gryllus bimaculatus*, *Helicoverpa armigera*, *Lepisma hesperum*, *Locusta migratoria*, *Oncopeltus asiaticus*, *Periplaneta americana* , *Plutella xylostella*, *Schistocerca gregaria*, *Spodoptera exigua* , *Tenebrio molitor* , *Thermobia madens*, *Tribolium castaneum* , and *Zootermopsis nevadensis* . Phylogenetic trees were built to investigate genes associated with environmental adaptation. Data S1–S15 contain all of the amino acid sequences that were used in this work. Multiple sequence alignment was performed using the Muscle program, alignment results were pruned using TrimAl, and the amino acid replacement model was automatically screened using IQ‐tree to create maximum likelihood (ML) trees (Zhou, Zhou, et al. 2023). These trees were visualized using FigTree v1.4.2 (Rambaut 2012) and the “One Step Build an ML Tree” functionality of TBtools (Zhu et al. 2022). The genes for the comparison between *G. sinensis* and *G. jilina* were mapped using Prism 8.0 (GraphPad, Boston, MA, USA).

### 2.6 RNA‐Seq Analysis With Gene Heatmap Visualization

Bowtie (Langmead et al. 2009) was used to align the sequencing reads with the Unigenes dataset, and RSEM (Li and Dewey 2011) was used to assess the expression levels of the reads. The expression abundance of the relevant gene transcripts was represented by the fragments per kilobase of exon model per million mapped fragments (FPKM) (Leitch et al. 2015). Heatmaps of these differentially expressed genes were generated using the clustering heatmap tool (https://hiplot.com.cn/cloud‐tool/drawing‐tool/detail 106) on the basis of Log_10_[FPKM + 1] values. The mean of three biological replicates (where each repetition is a separate entity) is called the FPKM in this case. Data S16 contains a presentation of all the raw FPKM data used in this investigation.

## 3 Results

### 3.1 Tissue‐Specific Transcriptome of *G. sinensis*

A minimum of 5.79 Gb of effective sequencing data was acquired from each of the six samples, for a total of 75.75 Gb. Table S1 shows that there were 253,332,843 clean read pairs and 66,131 unigenes with an N50 of 2200 for unigenes (Table S2 and Figure S1). BUSCO analysis of the transcriptome data yielded the following results: 84.2% (S: 83.1%, D: 1.1%), F: 8.1%, M: 7.7%, *n*: 1658. Similarity searches were carried out for sequence annotation against the following nine public databases: NR, Swiss‐Prot, TrEMBL, KEGG, COG, KOG, GO, eggNOG, and Pfam. A total of 19,147 unigenes were successfully annotated in various databases according to the results (Table S3).

### 3.2 Chemosensory Gene Identification and Homology Analysis

**FIGURE 2:**
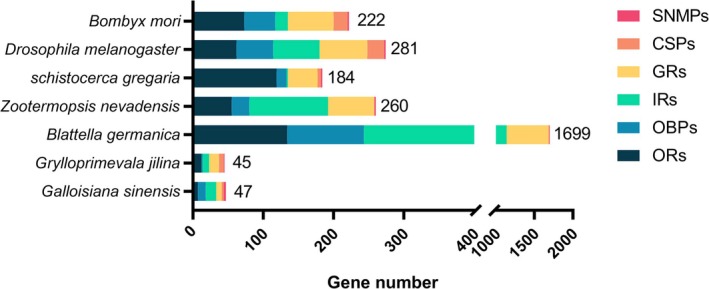
Comparison of the chemosensory genes of various animals.

**FIGURE 3:**
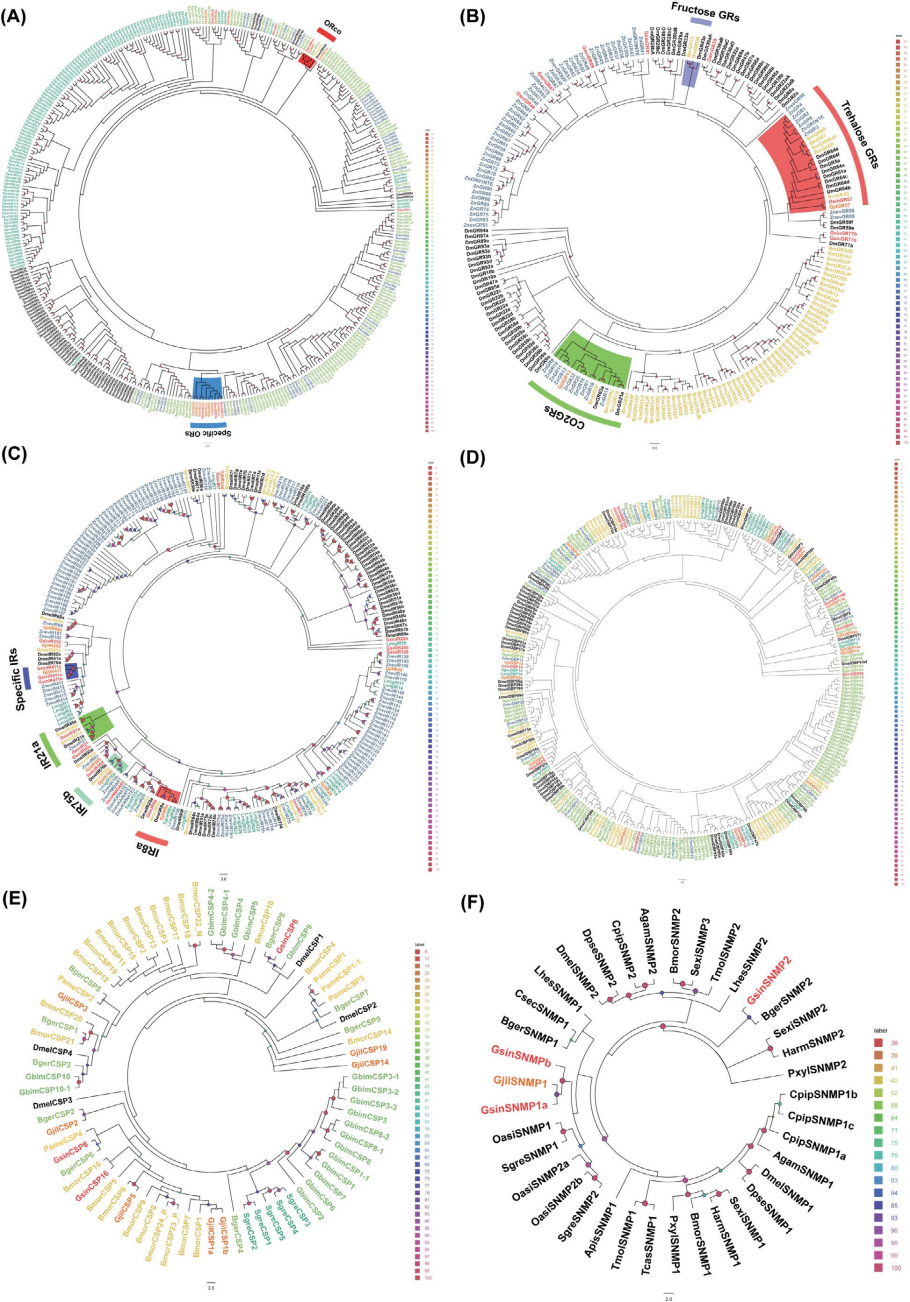
Chemosensory genes from *Galloisiana sinensis* , *Grylloprimevala jilina*, and other representative insect species were analyzed for homology. (A) Analysis of homology between *G. sinensis* ORs and those from other representative insect species. Gjil (Zhou, Zhou, et al. 2023): *G. jilina*; Bger (Robertson et al. 2018): *Blattella germanica* ; Dmel (Couto et al. 2005): *Drosophila melanogaster* ; Sgre (Pregitzer et al. 2017): *Schistocerca gregaria*; Znev (Terrapon et al. 2014): *Zootermopsis nevadensis* ; Pame (Chen et al. 2016): *Periplaneta americana*. (B) Analysis of the homology between *G. sinensis* GRs and those from other representative insect species. Gjil (Zhou, Zhou, et al. 2023): *G. jilina*; Bger (Robertson et al. 2018): *B. germanica* ; Dmel (Robertson et al. 2003): *D. melanogaster* ; Bmor (Guo et al. 2017): *Bombyx mori* ; Znev (Terrapon et al. 2014): *Z. nevadensis*. (C) Analysis of the homology between *G. sinensis* IRs and those from other representative insect species. Gjil (Zhou, Zhou, et al. 2023): *G. jilina*; Lmig (Wang et al. 2015): *Locusta migratoria*; Dmel (Croset et al. 2010): *D. melanogaster* ; Bmor (Olivier et al. 2011): *B. mori* ; Pame (Chen et al. 2016): *P. americana* ; Sgre (Guo et al. 2014): *S. gregaria* ; Znev (Terrapon et al. 2014): *Z. nevadensis*. (D) Analysis of the homology between *G. sinensis* OBPs and those from other representative insect species. Gjil (Zhou, Zhou, et al. 2023): *G. jilina*; Bger (Niu et al. 2016): *B. germanica* ; Lmig (Li et al. 2018): *L. migratoria*; Dmel (Vieira and Rozas 2011): *D. melanogaster* ; Bmor (Gong et al. 2009): *B. mori* ; Pame (Xu et al. 2009): *P. americana* ; Sgre (Yang et al. 2012): *S. gregaria* ; Znev (Terrapon et al. 2014): *Z. nevadensis* ; and Gbim (Xu et al. 2009): *Gryllus bimaculatus*. (E) Analysis of the homology between *G. sinensis* OBPs and those from other representative insect species. Gjil (Zhou, Zhou, et al. 2023): *G. jilina*; Bger (Niu et al. 2016): *B. germanica* ; Dmel (Vieira and Rozas 2011): *D. melanogaster* ; Bmor (Gong et al. 2007): *B. mori* ; Pame (Xu et al. 2009): *P. americana* ; Sgre (Angeli et al. 1999): *S. gregaria* ; and Gbim (Xu et al. 2009): *G. bimaculatus*. (F) Analysis of the homology between *G. sinensis* OBPs and those from other representative insect species. Gjil (Zhou, Zhou, et al. 2023): *G. jilina*; Bger (Niu et al. 2016): *B. germanica* ; Dmel (Vogt et al. 2009): *D. melanogaster* ; Bmor (Vogt et al. 2009): *B. mori* ; Sgre (Jiang et al. 2016): *S. gregaria* ; Dpse (Vogt et al. 2009): *Drosophila pseudoobscura* ; Cpip (Vogt et al. 2009): *Culex pipiens* ; Oasi (Zhou et al. 2019): *Oedaleus asiaticus*; Sexi (Zhang et al. 2015): *Spodoptera exigua* ; Pxyl (Li and Qin 2011): *Plutella xylostella*; Harm (Vogt et al. 2009): *Helicoverpa armigera*; Tcas (Vogt et al. 2009): *Tribolium castaneum* ; Agam (Vogt et al. 2009): *Anopheles gambiae* ; Tmol (Liu et al. 2015): *Tenebrio molitor* ; Lhes (Tassone et al. 2016): *Lygus hesperus* ; Apis (Purandare and Brisson 2020): *Acyrthosiphon pisum* ; Csec (He et al. 2022): *Cryptotermes secundus*.

The number of chemosensory genes found in *G. sinensis* (45; 12 ORs, 14 OBPs, 7 CSPs, 9 IRs, 2 GRs, and 1 SNMP) was substantially lower than that found in other species and comparable to the 45 chemosensory genes found in *G. jilina* (Figure 2). Six chemosensory gene families were screened from the *G. sinensis* transcriptome sequence. A single branch contained the homologous genes *GsinORCO*, *GjilORCO*, and others (Table S4 and Figure 3A). Among their ORs, *G. sinensis* and *G. jilina* each displayed a distinct evolutionary lineage (blue). As shown in Table S5 and Figure 3B, eight GRs were identified, and their homology with known insects ranged from 24.18% to 76.39%. The similarity of the 15 discovered IRs ranged from 28.75% to 98.10%. IR8a formed a cluster along with *GjilIR8a*, *DmelIR8a*, *BmorIR8a*, *SgreIR8a*, and *LmigIR8a*. In the 93a family, *GsinIR93a* clustered with *GjilIR93a*, *DmelIR93a*, *BmorIR93a*, and *ZnevIR93*. Table S6 and Figure 3C show that *GsinIR411a* and *GsinIR411b*, as well as *GjilIR411*, were grouped in separate branches. The separate branches *GsinOBP30a* and *GsinOBP30b* clustered together. Additionally, *GsinOBP89* and *GsinOBP89* (Table S7 and Figure 3D) did not cluster with other species, such as *G. jilina*. *GsinCSP6*, *GsinCSP8*, *GsinCSP16*, and *GsinCSP14* did not group with other species, such as *G. jilina*. In a single clade, no GsinCSP gene was strongly grouped with the GjinCSP gene (Table S8 and Figure 3E). *G. sinensis* possesses SNMP2, in contrast to *G. jilina* (Table S9 and Figure 3F).

### 3.3 Vision‐Related Gene Identification and Homology Analysis

**FIGURE 4:**
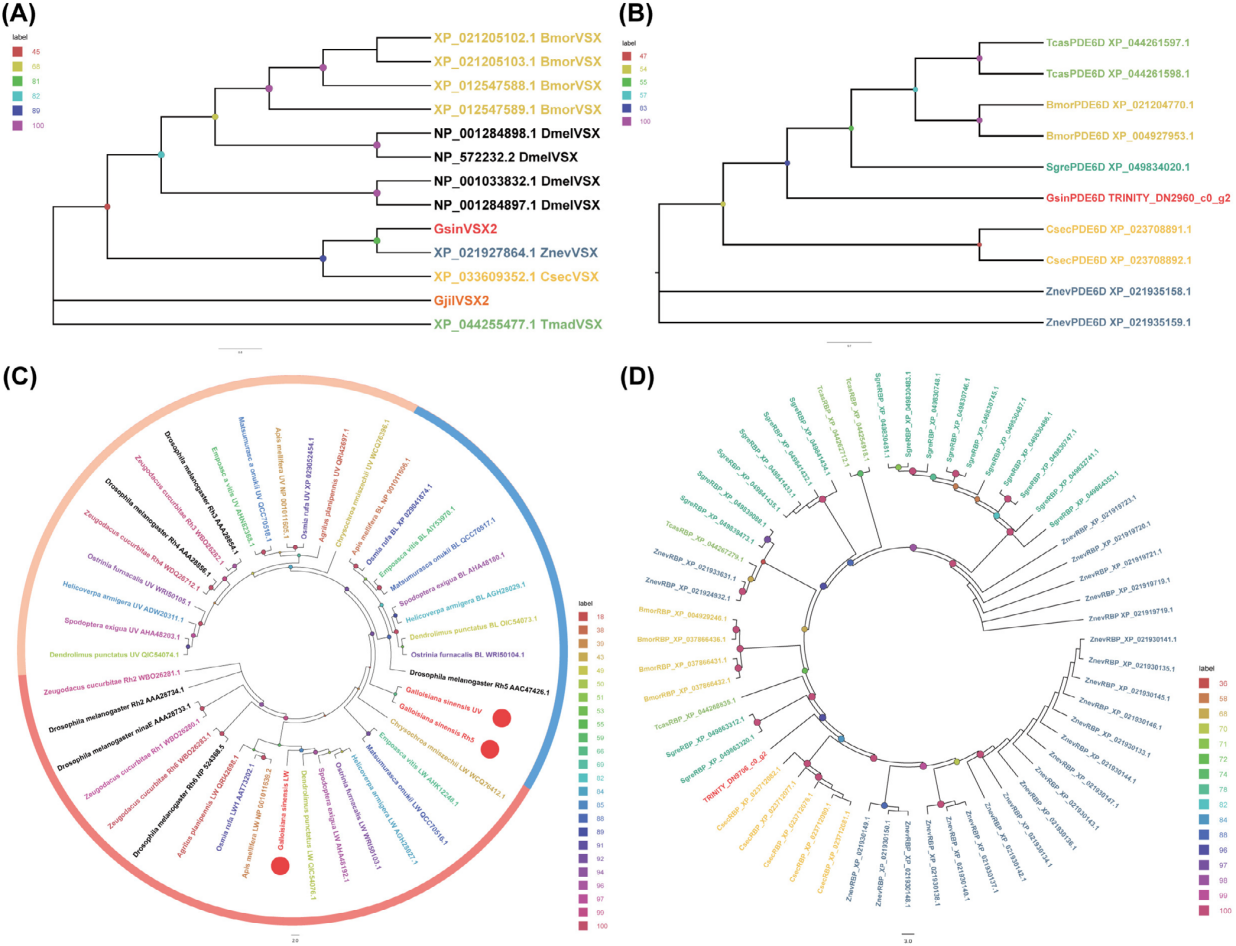
Homology analysis of vision‐related genes from *Galloisiana sinensis* and other representative insect species. (A) Analysis of homology between VSXs from *G. sinensis* , *Grylloprimevala jilina*, and other representative species of insects. Gjil: *G. jilina* (Zhou, Zhou, et al. 2023); Tmad (GCF_015345945.1): *Tribolium madens*; Znev (GCF_000696155.1): *Zootermopsis nevadensis* ; Dmel (GCF_000001215.4): *Drosophila melanogaster* ; Csec (GCF_002891405.2): *Cryptotermes secundus*; Bmor (GCF_014905235.1): *Bombyx mori* . (B) Analysis of the homology between PDE6Ds from *G. sinensis* and other representative species of insects. Bmor (GCF_014905235.1): *B. mori* ; Csec (GCF_002891405.2): *C. secundus*; Dmel (GCF_000001215.4): *D. melanogaster* ; Tmad (GCF_015345945.1): *T. madens*; Znev (GCF_000696155.1): *Z. nevadensis* ; Sgre (GCF_023897955.1): *Schistocerca gregaria*. (C) Analysis of the homology between opsins from *G. sinensis* and other representative species of insects. These include Diptera, Hemiptera, Hymenoptera, Coleoptera, and Lepidoptera insects (Huang et al. 2024). (D) Analysis of the homology between opsins from *G. sinensis* and other representative species of insects. Bmor (GCF_014905235.1): *B. mori* ; Csec (GCF_002891405.2): *C. secundus*; Dmel (GCF_000001215.4): *D. melanogaster* ; Tmad (GCF_015345945.1): *T. madens*; Znev (GCF_000696155.1): *Z. nevadensis* ; Sgre (GCF_023897955.1): *S. gregaria* .

We discovered more vision‐related genes in *G. sinensis* than in *G. jilina*, where only one gene was shown to be involved in vision. Using *G. sinensis* transcriptome sequencing, four gene families relevant to vision were identified. *GsinVSX2*, a VSX gene, was identified. *GsinVSX2* and *GjilVSX2* belong to different clades, and *GjilVSX2* is a fairly independent clade (Table S10 and Figure 4A). The maximum similarity with other homologs was 73.19%, as determined by BLAST. Three opsins exhibited similarity between 39.81% and 85.90% with known insects, as shown in Table S11 and Figure 4B. BLAST analysis confirmed that GsinPDE6D exhibited the highest similarity (92.52%) with other insect homologs (Table S12 and Figure 4C). A single RBP, GsinRBP, was identified, showing 61.09% sequence similarity with its closest homologs by BLAST analysis (Table S13 and Figure 4D).

### 3.4 Temperature Adaptation‐Related Gene Identification and Homology Analysis

**FIGURE 5:**
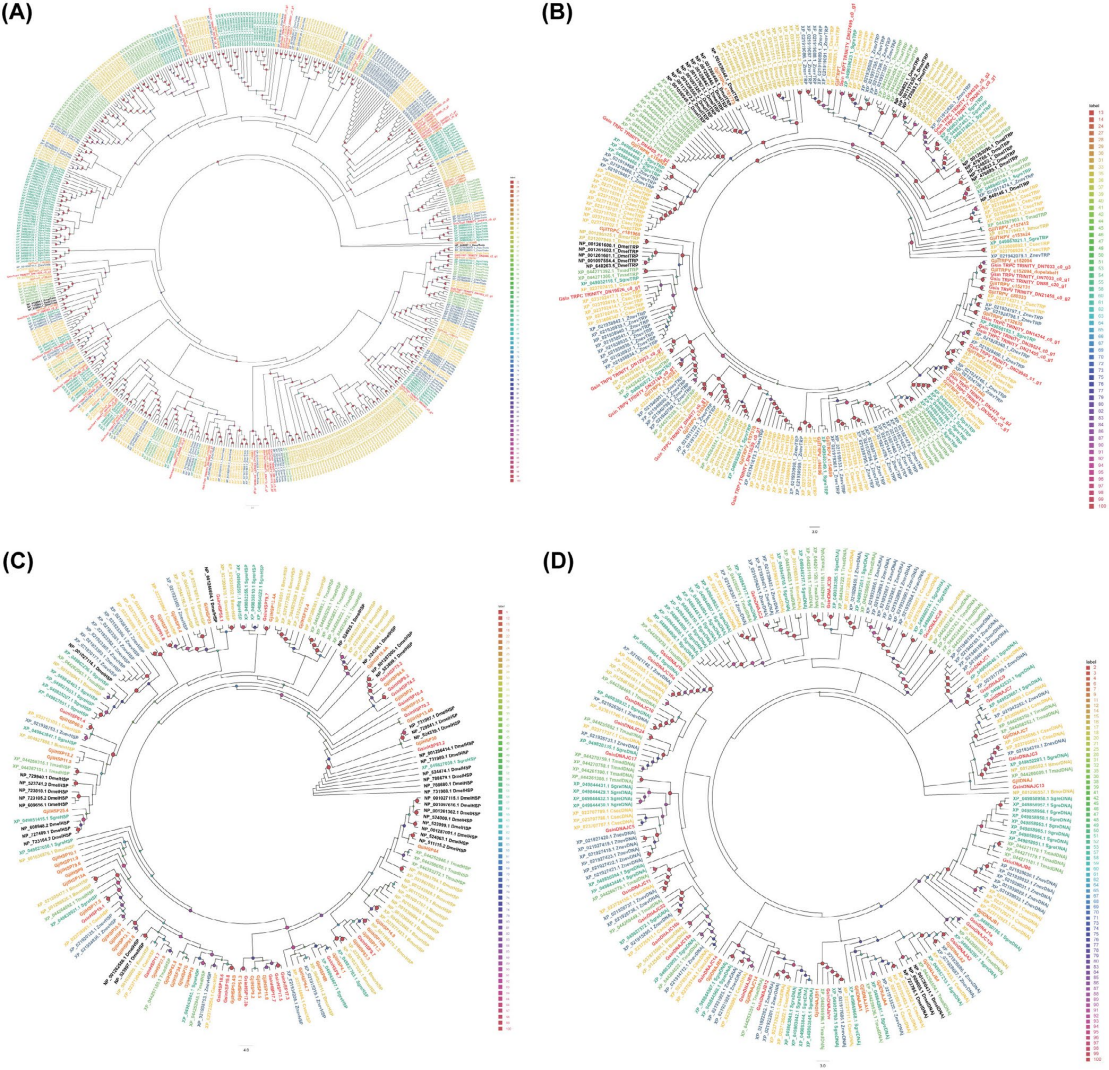
Temperature adaptation‐related genes from *Galloisiana sinensis* , *Grylloprimevala jilina*, and other representative insect species were analyzed using homology. (A) Homology analysis of Trets from *G. sinensis* , *G. jilina*, and other representative insect species. Gjil (Zhou, Zhou, et al. 2023): *G. jilina*, Bmor (GCF_014905235.1): *Bombyx mori* , Csec (GCF_002891405.2): *Cryptotermes secundus*, Dmel (GCF_000001215.4): *Drosophila melanogaster* , Sgre (GCF_023897955.1): *Schistocerca gregaria*, Tmad (GCF_015345945.1): *Tribolium madens*, Znev (GCF_000696155.1): *Zootermopsis nevadensis* . (B) Homology analysis of TRPs from *G. sinensis* , *G. jilina*, and other representative insect species. Gjil (Zhou, Zhou, et al. 2023): *G. jilina*, Bmor (GCF_014905235.1): *B. mori* , Csec (GCF_002891405.2): *C. secundus*, Dmel (GCF_000001215.4): *D. melanogaster* , Sgre (GCF_023897955.1): *S. gregaria* , Tmad (GCF_015345945.1): *T. madens*, Znev (GCF_000696155.1): *Z. nevadensis* . (C) Homology analysis of HSPs from *G. sinensis* , *G. jilina*, and other representative insect species. Gjil (Zhou, Zhou, et al. 2023): *G. jilina*, Bmor (GCF_014905235.1): *B. mori* , Csec (GCF_002891405.2): *C. secundus*, Dmel (GCF_000001215.4): *D. melanogaster* , Sgre (GCF_023897955.1): *S. gregaria* , Tmad (GCF_015345945.1): *T. madens*, Znev (GCF_000696155.1): *Z. nevadensis* . (D) Homology analysis of DnaJs from *G. sinensis* , *G. jilina*, and other representative insect species. Gjil (Zhou, Zhou, et al. 2023): *G. jilina*, Bmor (GCF_014905235.1): *B. mori* , Csec (GCF_002891405.2): *C. secundus*, Dmel (GCF_000001215.4): *D. melanogaster* , Sgre (GCF_023897955.1): *S. gregaria* , Tmad (GCF_015345945.1): *T. madens*, Znev (GCF_000696155.1): *Z. nevadensis* .

Four gene families associated with temperature adaptation were examined. A total of 26 Trets were found, showing 39.37%–84.39% similarity with each other and with other species. Except for *GsinTret1‐TRINITY_DN6516*, GsinTret1s clustered with GjilTret1s (Table S14 and Figure 5A). A total of 19 TRP genes were identified. Most of the GsinTret1s and GjilTret1s were homologous. Moreover, GsinTret1 and GjilTret1 formed one large cluster in the phylogenetic tree. Twenty HSPs were identified, which formed five large clusters in the phylogenetic tree (Table S15 and Figure 5B). A total of 26 DnaJs were identified, which were the least comparable to those of other insects (54.14%) (Table S16 and Figure 5C). Most of the GsinHSPs and GjilHSPs were homologous, such as *GsinHsp61.4* and *GjilHsp60.9* and Gsin*Hsp17.5* and *GjilHsp19.1*. Moreover, most GsinDnaJs were less homologous to GjilDnaJs, with only a small number of genes clustered in one clade (Table S17 and Figure 5D).

### 3.5 Identification and Homology Analysis of Genes Linked to Wing Morph Differentiation

**FIGURE 6:**
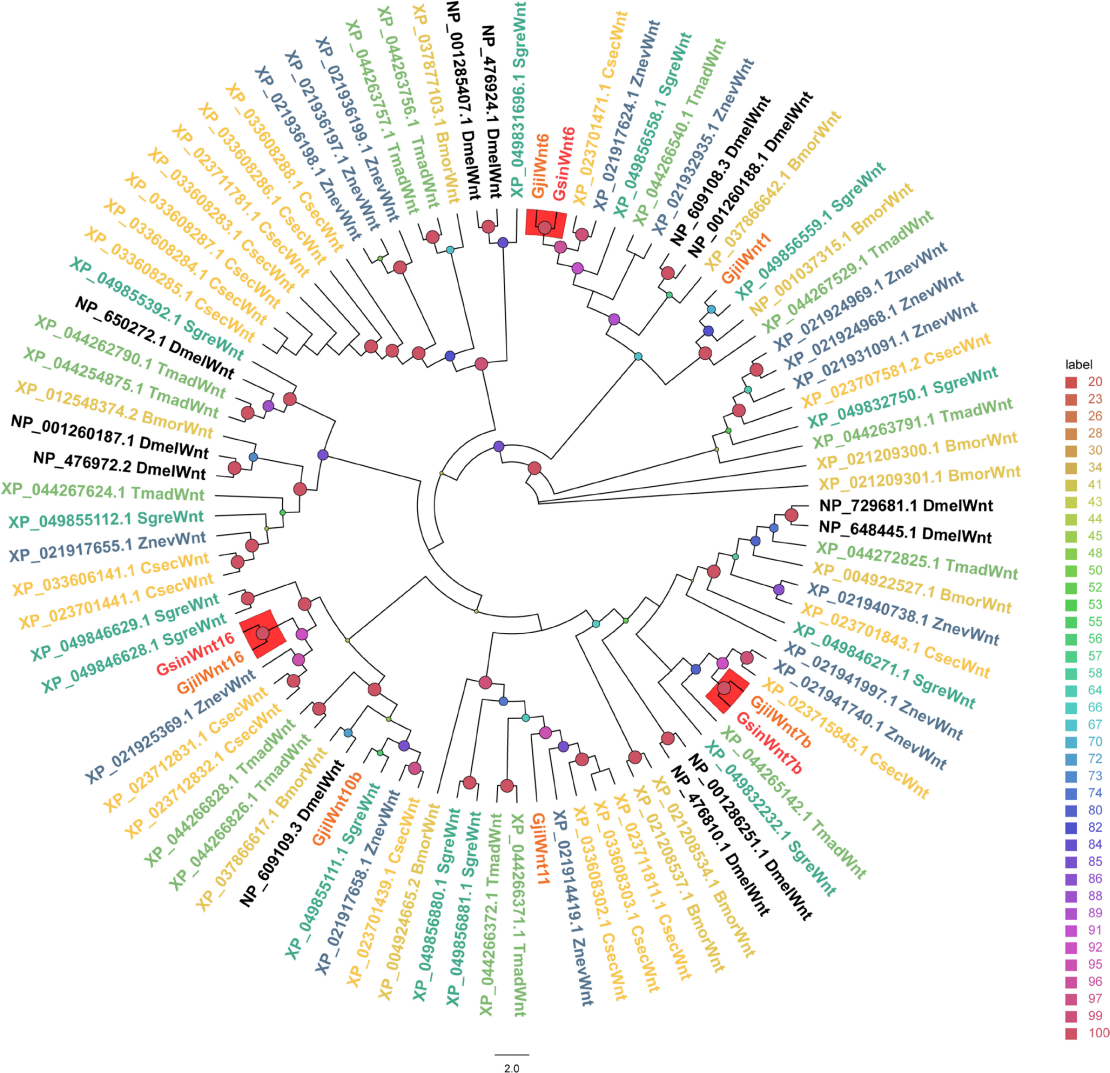
Wnts from *Galloisiana sinensis* and other typical insect species were analyzed for homology. Gjil (Zhou, Zhou, et al. 2023): *Grylloprimevala jilina*, Bmor (GCF_014905235.1): *Bombyx mori* , Csec (GCF_002891405.2): *Cryptotermes secundus*, Dmel (GCF_000001215.4): *Drosophila melanogaster* , Sgre (GCF_023897955.1): *Schistocerca gregaria*, Tmad (GCF_015345945.1): *Tribolium madens*, Znev (GCF_000696155.1): *Zootermopsis nevadensis* .

Table S18 and Figure 6 show that three Wnt genes were 79.89%–83.33% similar to those of other species. *GsinWnt6* and *GjilWnt6* clustered in one clade; *GsinWnt7b* and *GjilWnt7b* clustered in one clade; and *GsinWnt16* and *GjilWnt16* clustered in one clade. Genes homologous to *GjilWnt1*, *GjilWnt10b*, and *GjilWnt11* were not found in *G. sinensis* .

### 3.6 Analysis of Gene Number and Expression in *G. sinensis*

**FIGURE 7:**
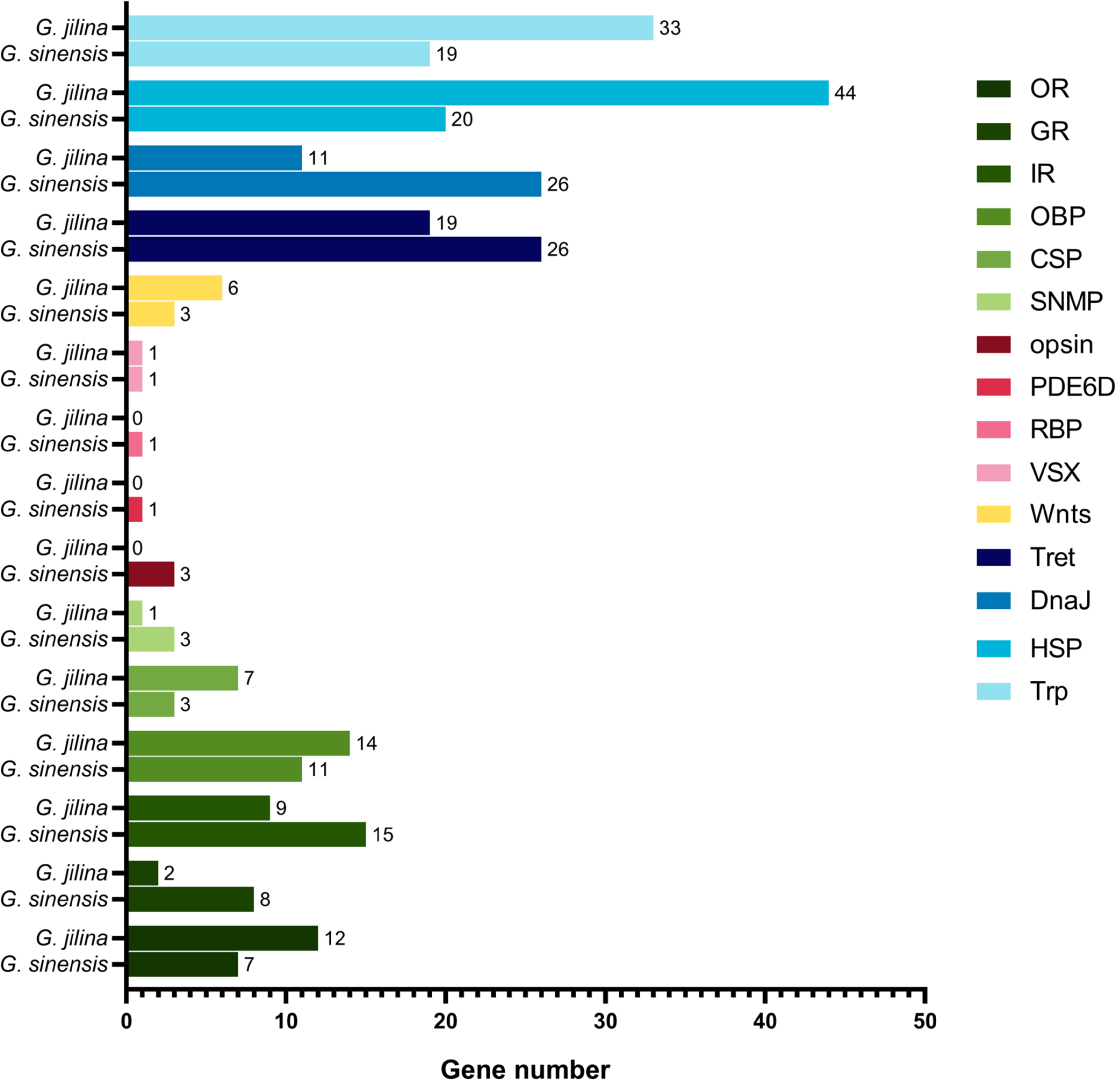
Genes linked to environmental adaptability in *Grylloprimevala jilina* and *Galloisiana sinensis* were compared.

Compared with *G. jilina*, *G. sinensis* possesses fewer ORs, OBPs, and CSPs but has a greater number of GRs, IRs, and SNMPs. *G. sinensis* is endowed with opsin, PDE6D, and RBP genes, which are absent in *G. jilina*. Moreover, *G. sinensis* presented a reduced number of HSPs and TRPs relative to *G. jilina* but compensated for the increased presence of DnaJs and Trets. Notably, *G. jilina* harbors a greater quantity of Wnt genes than *G. sinensis* (Figure 7). On the basis of the findings of the FPKM values from transcriptome sequencing of four tissue samples (heads, thoraxes, abdomens, and legs), chemosensory genes, notably OBPs and CSPs, are expressed primarily in the head (Figure S2). Among the vision‐related genes, VSX expression was not detected in the legs, and UV expression was not detected in the thorax (Figure S3). Temperature adaptation‐related genes are expressed throughout the body (Figure S4). With respect to wing differentiation genes, Wnt7b expression was not detected in the thorax, whereas Wnt6 and Wnt16 were detected in all body parts (Figure S5).

## 4 Discussion

Compared with other insects, such as *D. melanogaster* , *B. mori* , and *Z. nevadensis* , *G. sinensis* and *G. jilina* present fewer chemosensory genes, totaling 47 and 45 genes, respectively (Robertson et al. 2003, 2018; Vogt et al. 2009; Croset et al. 2010; Vieira and Rozas 2011; Terrapon et al. 2014; Niu et al. 2016; Venthur and Zhou 2018). Studies have revealed that diverse insect odorant receptors (ORs) emerge after the development of wings (Missbach et al. 2014) and that chemosensory receptors seem to degrade when chemosensory dependence decreases (Gilad et al. 2003), suggesting that a reduction in the expression of chemosensory genes might be associated with wing loss. Compared with *G. jilina*, *G. sinensis* has fewer ORs, OBPs, and CSPs, and more GRs, IRs, and SNMPs; these differences in chemosensory genes may be linked to environmental deviations, with *G. jilina* being a true cave dweller and *G. sinensis* inhabiting a relatively food‐abundant environment (Zhou, Zhou, et al. 2023). Comparative analysis of ORs and OBPs between *G. sinensis* and *G. jilina* revealed considerable disparities, with no homologous GjilOR counterpart to *GsinOR1* and no GjilOBP counterpart in the *GsinOBP30a* clade. These differences seemingly correlate with their distinct habitats: *G. sinensis* lives on barren hill slopes, and *G. jilina* lives in caves (Zhou, Chen, et al. 2023). However, both species share some ORs and OBPs within the same phylogenetic branches, for example, *GsinOR39*, *GjilOR39a*, *GsinOBP12*, and *GjilOBP12*, indicating that this gene conservation is potentially tied to intraspecies communication. Notably, *G. jilina* lacks homologs for GsinGR41, *GsinGR46*, *GsinGR47*, and *GsinGR49*, which are more similar to *Z. nevadensis* GRs (Terrapon et al. 2014), suggesting potential degradation due to the impoverished cave environment. In *G. sinensis* , no CO_2_‐sensitive GR was detected (Yang et al. 2024), possibly because of gene loss or unsequenced transcripts. Given that GR64 in *Drosophila* detects sugar (Slone et al. 2007; Montell 2009), *GsinGR57* and *GjilGR57*, which belong to the same lineage, are inferred to be sugar receptors, implying that both species can taste sugar. Both species share a specialized IR branch, with IR411 appearing duplicated in *G. sinensis* . IR8a is known to form functional olfactory receptor complexes in flies and mosquitoes (Ai et al. 2013; Raji et al. 2019; Zhang et al. 2019) and mediates cold sensation via IR21a (Ni et al. 2016; Greppi et al. 2020), functions likely conserved in both Grylloblattodea species. IR75b senses acids in some insects (Prieto‐Godino et al. 2021), yet no IR75b homolog was found in *G. sinensis* , suggesting a lack of this function or detection through alternative IRs or absent sequences. No CSPs from the two species clustered together, indicating rapid evolution of CSPs compared with other chemosensory genes. SNMP1 is typically associated with pheromone reception in antennae (Forstner et al. 2008), whereas SNMP2 is more broadly expressed (Vogt et al. 2009), akin to what is observed in *G. sinensis* , which possesses two SNMP1 copies, one of which (SNMP1a) is most similar to GjilSNMP1.

In addition, the wing degeneration observed in Grylloblattodea may be partially attributed to nutritional constraints and trophic specialization associated with its subterranean lifestyle. Wing musculature becomes maladaptive when dietary energy intake is chronically low. This aligns with the resource allocation trade‐off model, where limited nutrients are redirected to reproduction and somatic maintenance at the expense of flight structures (Harrison and Roberts 2000).

*Grylloprimevala jilina* is a true cave insect, in which we previously identified only the VSX gene in our research (Zhou, Zhou, et al. 2023). In contrast, in *G. sinensis* , which inhabits a sunlit environment, a homology analysis of VSX revealed that the *GjilVSX* gene does not cluster with those of other species. Instead, *GsinVSX* shows greater homology with *Z. nevadensis* VSX and appears more akin to a gene that plays a vital role in visual system development than does *GjilVSX* (Posnien et al. 2023). Furthermore, the identification of a blue light‐sensitive opsin and long wavelength‐sensitive opsin in *G. sinensis* suggests that it can perceive light of different wavelengths (Huang et al. 2024). Additionally, the detection of the PDE6D gene and RBP in *G. sinensis* is noteworthy; PDE6D is crucial in modulating the light sensitivity of retinal receptor cells (Link and Collery 2015), whereas RBP ensures an adequate supply of retinol to the retina (Pelosi et al. 2018), collectively providing evidence for the existence of a more sophisticated visual system in *G. sinensis* . Compared with *H. armigera* (Lepidoptera), *D. melanogaster* (Diptera), *Apis mellifera* (Hymenoptera), *L. migratoria* (Orthoptera), *B. germanica* (Blattodea), and *G. sinensis* presented similar numbers of *GsinVSX*, *GsinPDE6D*, and *GsinRBP* genes but a notably reduced repertoire of opsin genes (Hardie and Padinjat 2001; Briscoe et al. 2010; Feuda et al. 2012; Guignard et al. 2022). This stark reduction in opsin diversity—compared with 6–8 in Lepidoptera, 5–7 in Diptera, 4–6 in Hymenoptera, 3–4 in Orthoptera, and 3–4 in Blattodea—suggests a progressive degeneration of phototransduction capacity, likely driven by its extreme subterranean and low‐light habitat (Friedrich et al. 2011).

*G. sinensis* inhabits the northern wilderness of China, where temperature fluctuations exceed 50°C annually (Zhang et al. 2021), whereas *G. jilina* dwells in caves with a constant 16°C temperature (Kou 2019). This starkly contrasting habitat temperature appears to have resulted in *G. sinensis* possessing fewer HSPs and TRPs but more DnaJ proteins and Tret elements than *G. jilina* does –151 (Matsuura et al. 2009). Trets and HSPs play pivotal roles in insect responses to environmental stresses, such as thermal challenges (Zhou et al. 2022). Homology analyses revealed high similarity among most Trets, TRPs, and HSPs between the two species, for example, GsinHsp61.4 and GjilHsp60.9; however, a subset of genes presented low homology, including *GsinTret1 TRINITY_DN2446_c2_g1*, *GsinHSP56.1*, and *GjilHSP44*, which may have significant implications for understanding *G. sinensis* 's adaptation to temperature variation and the constant‐temperature lifestyle of *G. jilina*. As cochaperones of HSPs play crucial roles in their diversity (Faust et al. 2020), it is intriguing that DnaJs displayed poor homology between the two species, suggesting faster evolution of DnaJ genes than other temperature‐adaptive genes, likely driven by their environmental disparities. Compared with the gene counts observed in *B. mori* (Lepidoptera), *D. melanogaster* (Diptera), and *A. mellifera* (Hymenoptera), *G. sinensis* presented a reduced number of GsinHSPs. However, the numbers of GsinTRPs and GsinDnaJs are comparable to those in *B. mori* , *D. melanogaster* , and *A. mellifera* (Li et al. 2009; Kampinga and Craig 2010; Nguyen et al. 2016). The low HSP gene count in *G. sinensis* likely reflects adaptation to stable environments where temperature fluctuations are minimal. This reduces selective pressure for HSP‐mediated stress tolerance, as observed in other stenothermic species (Beasley‐Hall et al. 2022). Lepidoptera and Hymenoptera face broader thermal ranges due to diurnal/nocturnal activity or seasonal foraging, necessitating the expansion of the HSP family (Li et al. 2009; Nguyen et al. 2016).

The order Notoptera, to which cockroaches belong, has undergone wing degeneration (Schoville et al. 2021); nonetheless, we still identified wing development‐related Wnt genes in *G. sinensis* (Gracia‐Latorre et al. 2022). Interestingly, more Wnt genes are expressed in *G. jilina* than in *G. sinensis* , and all GsinWnt genes cluster together with their GjilWnt counterparts, suggesting that the process of wing regression might have occurred at a faster rate in *G. sinensis* .

In conclusion, this research elucidated chemosensory genes, temperature adaptation‐related genes, vision‐related genes, and winged morph differentiation‐related genes in *G. sinensis* . We performed a comparative analysis with their counterparts in *G. jilina* and reported that while both species present comparable reductions in the expression of chemosensory genes potentially tied to wing loss, nuanced distinctions exist. Specifically, *G. sinensis* presented a diminished count of ORs, OBPs, and CSPs, accompanied by an enrichment of GRs, IRs, and SNMPs, potentially for adaptation to its habitats. Furthermore, compared with *G. jilina*, *G. sinensis* possesses a greater number of vision‐related genes, endowing it with a more robust photoreceptive system. Compared with *G. jilina*, *G. sinensis* presents a reduced number of HSPs and TRPs but harbors increased quantities of DnaJ proteins, suggesting that these proteins may increase chaperone activity to cope with wider temperature fluctuations. Despite the overall loss of flight capacity in Notoptera, the persistence of Wnt genes is noted, with *G. sinensis* presenting a much smaller lifespan than *G. jilina*, which is indicative of more profound wing regression. However, owing to the limited sample size and the use of whole‐head tissue (rather than isolated antennae) for transcriptome sequencing, some poorly expressed chemosensory genes (e.g., specific ORs or OBPs) may have been undetected. The analysis of transcriptomic datasets can also inform management strategies for threatened wild populations. It is crucial to use transcriptomic data to maximize effective conservation and management efforts, especially for endangered insects. These results have important implications for understanding and conserving Grylloblattodea.

## Author Contributions

**Yanhan Zhou:** conceptualization (lead), investigation (lead), supervision (equal), validation (equal), writing – original draft (lead). **Kaipeng Zhang:** formal analysis (equal), methodology (lead), validation (equal), writing – original draft (equal), writing – review and editing (equal). **Luyao Yu:** formal analysis (equal), methodology (equal), validation (equal). **Yuxin Zhou:** formal analysis (equal), methodology (equal), validation (equal), writing – original draft (equal). **Yuetong Xiao:** formal analysis (equal), methodology (equal), validation (equal). **Lin Zhou:** formal analysis (equal), investigation (equal). **Xiaoyan Zhu:** formal analysis (equal), investigation (equal), validation (equal). **Taoqi Wang:** formal analysis (equal). **Qi Chen:** conceptualization (equal), project administration (lead), supervision (equal), writing – review and editing (equal). **Bingzhong Ren:** conceptualization (equal), project administration (equal), supervision (equal), writing – review and editing (equal).

## Conflicts of Interest

The authors declare no conflicts of interest.

## Supporting information


**Figures S1–S5:** ece372260‐sup‐0001‐FiguresS1‐S5.docx.

**Tables S1–S18:** ece372260‐sup‐0002‐TablesS1‐S18.doc.

**Data S1–S15:** ece372260‐sup‐0003‐DataS1‐S15.docx.

**Data S16:** ece372260‐sup‐0004‐DataS16.xlsx.

## Data Availability

All relevant data linked to the manuscript are available in the Supporting Information and the transcriptome data of *Galloisiana sinensis* has been uploaded to the National Genome Science Data Center, with the GSA number CRA027546.
