# Supplementary material for: Transcriptomic Analysis and Comparative Analysis of Gene Families Related to Environmental Adaptation in Two Grylloblattodea Species: Galloisiana sinensis and Grylloprimevala jilina
Source: Ecol Evol. 2025 Oct 7;15(10):e72260. doi: 10.1002/ece3.72260 (PMC12502051; doi:10.1002/ece3.72260)
Supplement: Supplementary file 1 — Figures S1–S5: ece372260‐sup‐0001‐FiguresS1‐S5.docx. [file ECE3-15-e72260-s002.docx]

**Supplementary materials.**


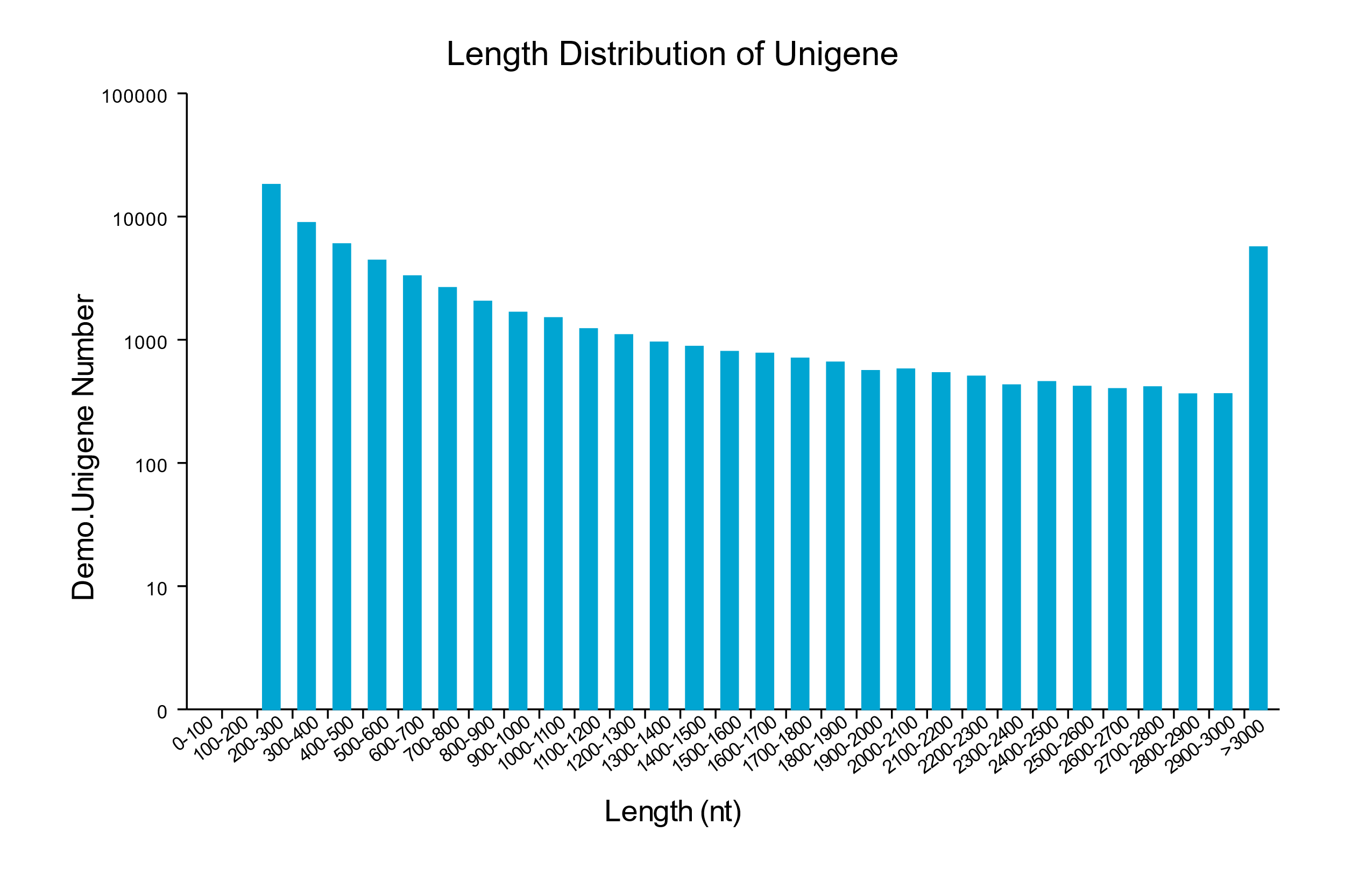


**Figure S1.** Size distribution of assembled Unigenes of *G. sinensis*. Length distribution of unigene after splicing.


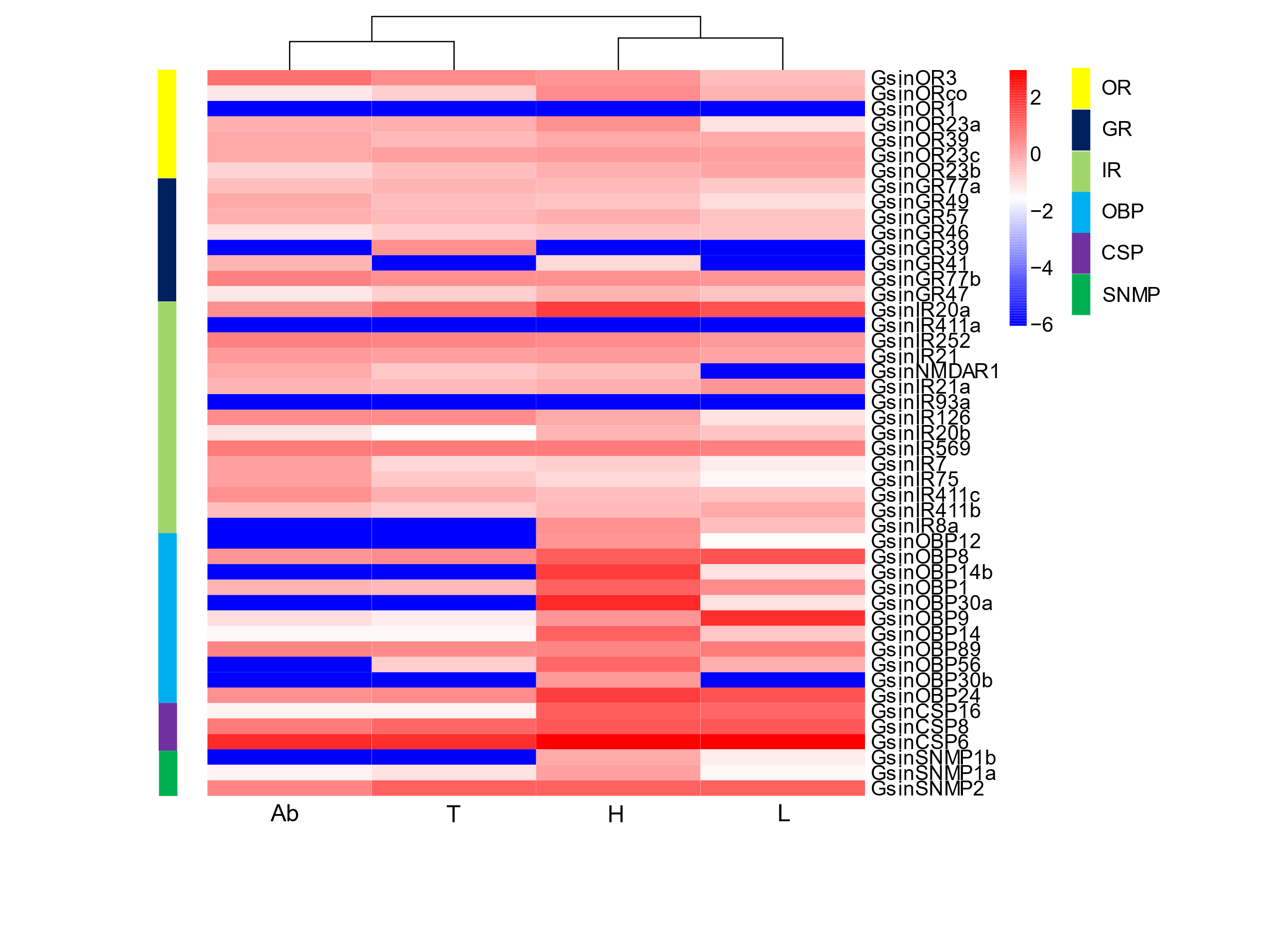


**Figure S2.** Expression patterns of chemosensory genes in *G. sinensis*. Each row and column represent a gene and specimen, respectively. The specimens are (H) heads, (T) thoraxes, (L) legs and (Ab) abdomens. The color gradient from red to green represents log10 (FPKM) values from large to small.


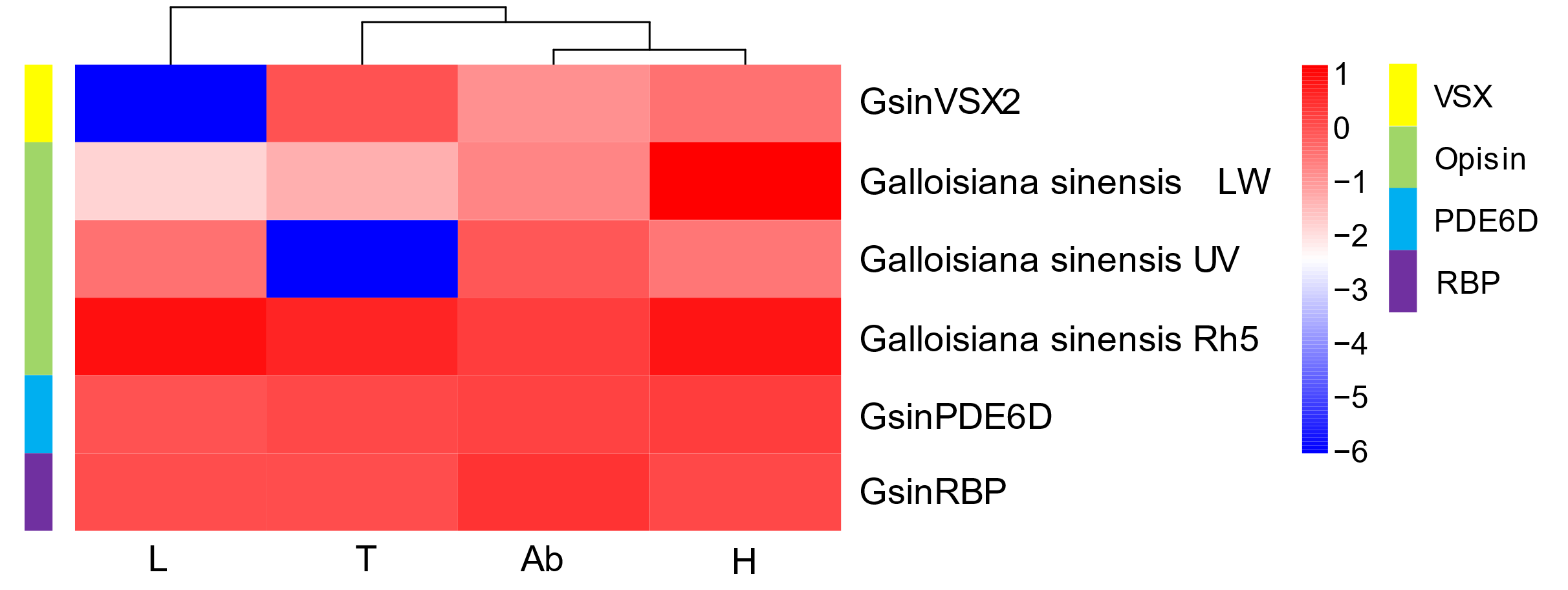


**Figure S3.** Expression patterns of vision-related genes in *G. sinensis*. Each row and column represent a gene and specimen, respectively. The specimens are (H) heads, (T) thoraxes, (L) legs and (Ab) abdomens. The color gradient from red to green represents log10 (FPKM) values from large to small.


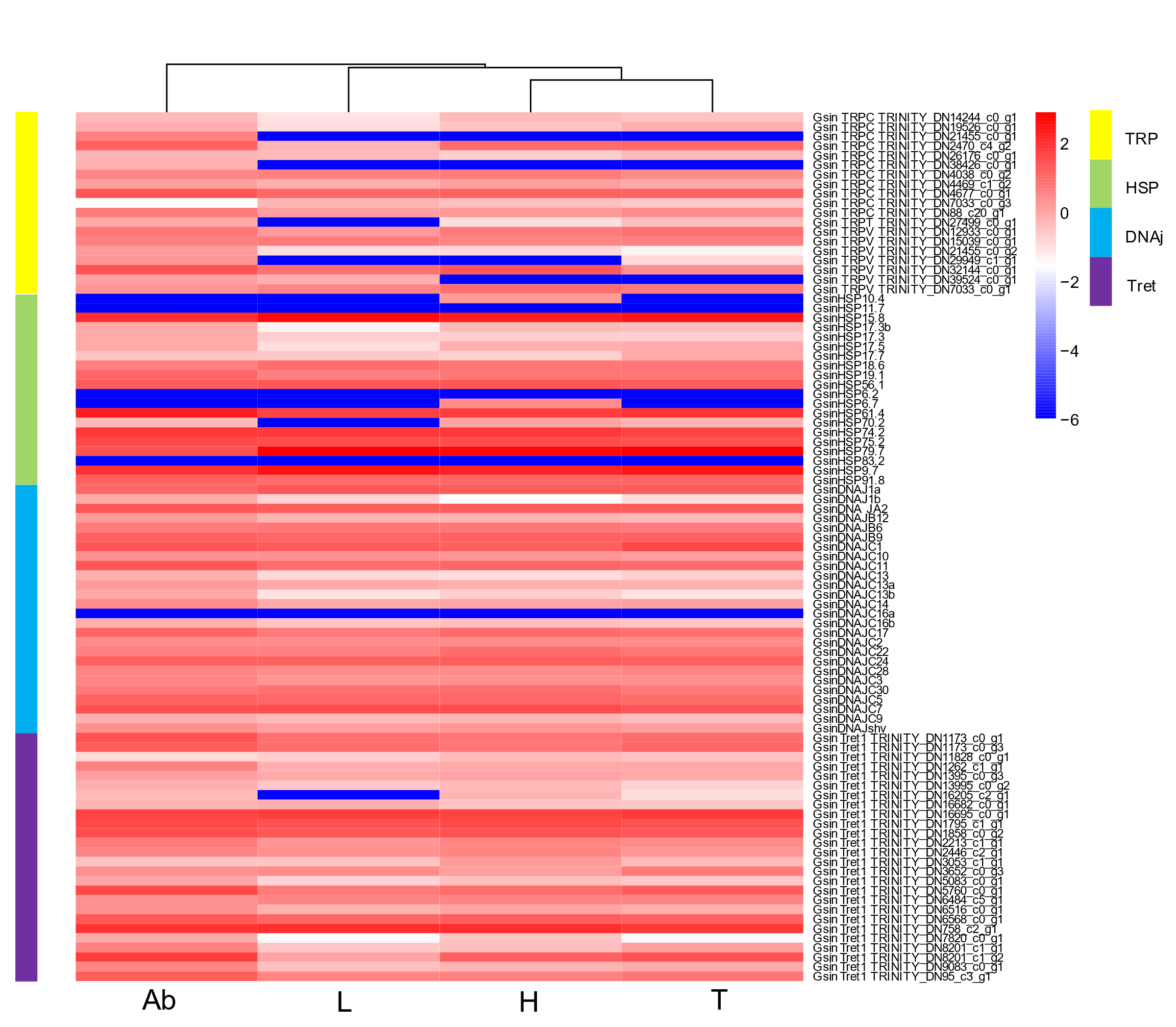


**Figure S4.** Expression patterns of temperature adaptation-related genes in *G. sinensis*. Each row represents a gene, and each column represents a specimen. The specimens are (H) heads, (T) thoraxes, (L) legs and (Ab) abdomens. The color gradient from red to green represents log10 (FPKM) values from large to small.


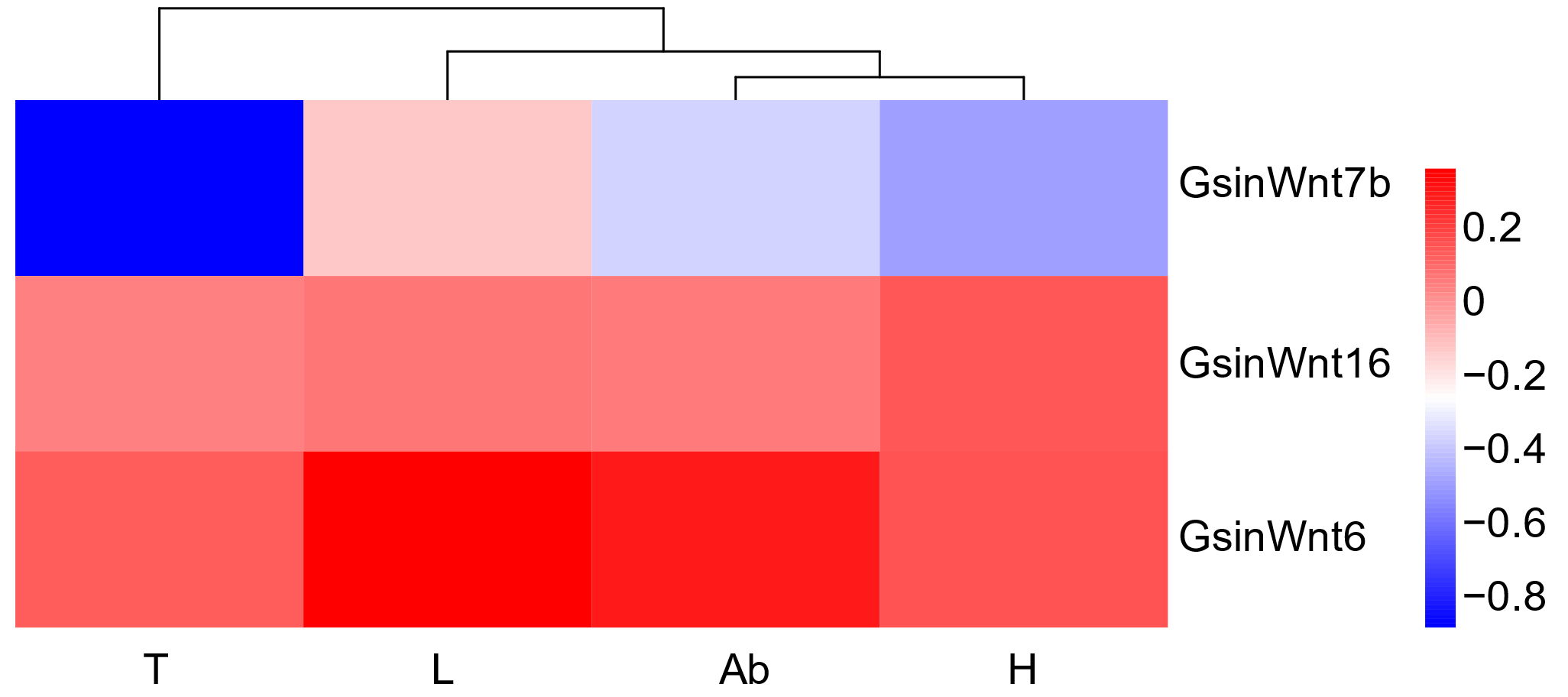


**Figure S5.** Expression patterns of winged morph differentiation-related genes in *G. sinensis.* Each row represents a gene, and each column represents a specimen. The specimens are (H) heads, (T) thoraxes, (L) legs and (Ab) abdomens. The color gradient from red to green represents log10 (FPKM) values from large to small.
