# Supplementary material for: Transcriptomic Analysis and Comparative Analysis of Gene Families Related to Environmental Adaptation in Two Grylloblattodea Species: Galloisiana sinensis and Grylloprimevala jilina
Source: Ecol Evol. 2025 Oct 7;15(10):e72260. doi: 10.1002/ece3.72260 (PMC12502051; doi:10.1002/ece3.72260)
Supplement: Supplementary file 2 — Tables S1–S18: ece372260‐sup‐0002‐TablesS1‐S18.doc. [file ECE3-15-e72260-s001.doc]

**Table S1 Clean data statistics**

| #SampleID | ReadSum | BaseSum | GC(%) | N(%) | Q20(%) | CycleQ20(%) | Q30(%) |
| --- | --- | --- | --- | --- | --- | --- | --- |
| Abdomen1 | 20490313 | 6129029666 | 44.82 | 0 | 98.19 | 100 | 94.81 |
| Abdomen2 | 21485000 | 6428707970 | 45.72 | 0 | 98.33 | 100 | 95.19 |
| Abdomen3 | 22415097 | 6704947194 | 44.67 | 0 | 98.23 | 100 | 94.98 |
| Head1 | 24070865 | 7192649310 | 40.45 | 0 | 98.14 | 100 | 94.76 |
| Head2 | 21240988 | 6352277162 | 42.15 | 0 | 98.11 | 100 | 94.67 |
| Head3 | 20438476 | 6111089818 | 40.13 | 0 | 98.04 | 100 | 94.46 |
| Leg1 | 19858969 | 5936414312 | 41.41 | 0 | 98.16 | 100 | 94.83 |
| Leg2 | 19380354 | 5793635302 | 43.89 | 0 | 97.44 | 100 | 93.25 |
| Leg3 | 21076264 | 6301237190 | 44.03 | 0 | 97.5 | 100 | 93.33 |
| Thorax1 | 20630956 | 6169076418 | 43.52 | 0 | 98.02 | 100 | 94.54 |
| Thorax2 | 21573700 | 6452614370 | 42.97 | 0 | 98.12 | 100 | 94.7 |
| Thorax3 | 20671861 | 6178312014 | 41.62 | 0 | 97.83 | 100 | 94 |

**Table S2 Statistics of assembly**

| Length Range | Transcript | Unigene |
| --- | --- | --- |
| 200-300 | 28,154(20.59%) | 18,087(27.35%) |
| 300-500 | 30,789(22.52%) | 14,837(22.44%) |
| 500-1000 | 29,004(21.22%) | 14,004(21.18%) |
| 1000-2000 | 22,955(16.79%) | 9,124(13.80%) |
| 2000+ | 25,803(18.87%) | 10,079(15.24%) |
| Total Number | 136,705 | 66,131 |
| Total Length | 167,286,650 | 70,416,962 |
| N50 Length | 2,370 | 2,200 |
| Mean Length | 1223.71 | 1064.81 |

**Table S3 Summary of unigene annotation**

| #Anno_Database | Annotated_Number | 300<=length<1000 | length>=1000 |
| --- | --- | --- | --- |
| COG_Annotation | 2,923 | 304 | 2,529 |
| GO_Annotation | 13,282 | 3,125 | 8,849 |
| KEGG_Annotation | 11,628 | 2,351 | 8,364 |
| KOG_Annotation | 8,873 | 1,494 | 6,808 |
| Pfam_Annotation | 12,060 | 2,388 | 8,911 |
| Swissprot_Annotation | 6,836 | 1,072 | 5,361 |
| TrEMBL_Annotation | 17,529 | 4,730 | 10,872 |
| eggNOG_Annotation | 13,579 | 3,212 | 9,130 |
| nr_Annotation | 17,579 | 4,750 | 10,909 |
| All_Annotated | 19,147 | 5,523 | 11,382 |

**Table S4. List of OR genes in *G. sinensis***

| **Number** | **Gene name** | **Unigene ID** | **Unigene ORF (aa)** | **BLASTx best hit** | **Query cover** | **E** | **Ident** | **The Length of "Best hit" (aa)** | **ID** |
| --- | --- | --- | --- | --- | --- | --- | --- | --- | --- |
| 1 | GsinOR3 | TRINITY_DN4902_c0_g1 | 450 | gustatory and odorant receptor 24-like isoform X2 [Zootermopsis nevadensis] | 88% | 6.00E-23 | 21.48% | 433 | XP_021913203.1 |
| 2 | GsinORco | TRINITY_DN25754_c0_g1 | 341 | odorant receptor coreceptor [Zootermopsis nevadensis] | 100% | 0 | 73.39% | 472 | XP_021933609.1 |
| 3 | GsinOR1 | TRINITY_DN19644_c0_g1 | 69 | odorant receptor Or2-like [Zootermopsis nevadensis] | 98% | 1.00E-10 | 47.06% | 437 | XP_021925437.1 |
| 4 | GsinOR23a | TRINITY_DN1472_c0_g2 | 110 | putative odorant receptor 71a [Zootermopsis nevadensis] | 89% | 1.00E-20 | 44.90% | 206 | XP_021936751.1 |
| 5 | GsinOR39 | TRINITY_DN6800_c1_g1 | 236 | olfactory receptor 24 [Adelphocoris lineolatus] | 74% | 9.00E-26 | 38.17% | 412 | APZ81446.1 |
| 6 | GsinOR23c | TRINITY_DN2691_c0_g1 | 436 | Odorant receptor 51a [Blattella germanica] | 83% | 2.00E-42 | 27.75% | 464 | PSN35482.1 |
| 7 | GsinOR23b | TRINITY_DN37695_c0_g1 | 51 | odorant receptor coreceptor-like [Cryptotermes secundus] | 86% | 1.00E-08 | 56.82% | 483 | XP_033611727.1 |

**Table S5. List of GR genes in *G. sinensis***

| **Number** | **Gene name** | **Unigene ID** | **Unigene ORF (aa)** | **BLASTx best hit** | **Query cover** | **E** | **Ident** | **The Length of "Best hit" (aa)** | **ID** |
| --- | --- | --- | --- | --- | --- | --- | --- | --- | --- |
| 1 | GsinGR77a | TRINITY_DN9742_c0_g1 | 89 | gustatory receptor 68a-like [Cryptotermes secundus] | 94% | 8.00E-13 | 41.67% | 465 | XP_033611065.1 |
| 2 | GsinGR49 | TRINITY_DN6899_c1_g2 | 373 | putative gustatory receptor 28a [Zootermopsis nevadensis] | 95% | 1.00E-16 | 24.18% | 439 | XP_021918944.1 |
| 3 | GsinGR57 | TRINITY_DN12141_c0_g1 | 229 | gustatory receptor for sugar taste 64f-like [Schistocerca nitens] | 99% | 8.00E-58 | 43.42% | 450 | XP_049799629.1 |
| 4 | GsinGR46 | TRINITY_DN15310_c0_g1 | 233 | glutamate receptor ionotropic, delta-2 isoform X3 [Cryptotermes secundus] | 99% | 1.00E-35 | 35.50% | 564 | XP_033607595.1 |
| 5 | GsinGR39 | TRINITY_DN18554_c0_g1 | 50 | gustatory receptor for sugar taste 64e-like [Macrosteles quadrilineatus] | 88% | 0.004 | 47.06% | 120 | XP_054259908.1 |
| 6 | GsinGR41 | TRINITY_DN19617_c0_g1 | 90 | putative gustatory receptor 28a isoform X4 [Zootermopsis nevadensis] | 80% | 3.00E-32 | 76.39% | 125 | XP_021920103.1 |
| 7 | GsinGR77b | TRINITY_DN558_c0_g2 | 673 | glutamate receptor 1 isoform X1 [Zootermopsis nevadensis] | 98% | 0 | 62.02% | 735 | XP_021922632.1 |
| 8 | GsinGR47 | TRINITY_DN20437_c0_g1 | 263 | putative gustatory receptor 28a [Cryptotermes secundus] | 93% | 5.00E-35 | 33.92% | 328 | XP_023702100.2 |

**Table S6. List of IR genes in *G. sinensis***

| **Number** | **Gene name** | **Unigene ID** | **Unigene ORF (aa)** | **BLASTx best hit** | **Query cover** | **E** | **Ident** | **The Length of "Best hit" (aa)** | **ID** |
| --- | --- | --- | --- | --- | --- | --- | --- | --- | --- |
| 1 | GsinIR20a | TRINITY_DN3716_c1_g1 | 92 | ionotropic receptor 25a isoform X1 [Zootermopsis nevadensis] | 100% | 0 | 83.59% | 935 | XP_021939206.1 |
| 2 | GsinIR411a | TRINITY_DN21096_c0_g1 | 54 | Ionotropic receptor 41a7 [Blattella germanica] | 90% | 0.001 | 46.94% | 686 | PSN32792.1 |
| 3 | GsinIR252 | TRINITY_DN2708_c2_g1 | 104 | ionotropic receptor 93a [Aricia agestis] | 53% | 2.00E-15 | 60.71% | 857 | XP_041979809.1 |
| 4 | GsinIR21 | TRINITY_DN12128_c0_g2 | 798 | ionotropic receptor 21a-like [Zootermopsis nevadensis] | 82% | 0 | 51.51% | 906 | XP_021925826.1 |
| 5 | GsinNMDAR1 | TRINITY_DN21740_c0_g1 | 227 | ionotropic receptor 40a-like isoform X2 [Homalodisca vitripennis] | 81% | 2.00E-50 | 50.54% | 587 | XP_046658718.1 |
| 6 | GsinIR21a | TRINITY_DN11520_c0_g1 | 194 | ionotropic receptor 93a [Zootermopsis nevadensis] | 88% | 2.00E-48 | 52.63% | 811 | XP_021926549.1 |
| 7 | GsinIR93a | TRINITY_DN21510_c0_g1 | 59 | Ionotropic receptor 75a [Gryllus bimaculatus] | 100% | 5.00E-20 | 62.71% | 243 | GLH05543.1 |
| 8 | GsinIR126 | TRINITY_DN191_c1_g1 | 615 | Ionotropic receptor 118 [Blattella germanica] | 96% | 2.00E-119 | 34.09% | 634 | PSN34589.1 |
| 9 | GsinIR20b | TRINITY_DN14280_c0_g1 | 302 | ionotropic receptor 25a.3 [Subpsaltria yangi] | 99% | 1.00E-115 | 56.33% | 927 | AXY87920.1 |
| 10 | GsinIR569 | TRINITY_DN16102_c0_g1 | 238 | ionotropic receptor 93a [Cryptotermes secundus] | 85% | 3.00E-73 | 54.90% | 736 | XP_033610759.1 |
| 11 | GsinIR7 | TRINITY_DN11856_c0_g1 | 408 | Ionotropic receptor 573 [Blattella germanica] | 96% | 1.00E-41 | 28.75% | 617 | PSN41928.1 |
| 12 | GsinIR75 | TRINITY_DN5857_c0_g1 | 151 | putative ionotropic receptor 25 [Conopomorpha sinensis] | 89% | 2.00E-47 | 48.89% | 234 | AXY83447.1 |
| 13 | GsinIR411c | TRINITY_DN27443_c0_g2 | 333 | Ionotropic receptor 41a9 [Blattella germanica] | 94% | 3.00E-58 | 36.28% | 676 | PSN57733.1 |
| 14 | GsinIR411b | TRINITY_DN38031_c0_g1 | 121 | Ionotropic receptor 41a12 [Blattella germanica] | 94% | 6.00E-19 | 41.23% | 704 | PSN57731.1 |
| 15 | GsinIR8a | TRINITY_DN38441_c0_g1 | 105 | ionotropic receptor 25a-like [Zootermopsis nevadensis] | 100% | 2.00E-63 | 98.10% | 815 | XP_021917623.1 |

**Table S7. List of OBP genes in *G. sinensis***

| **Number** | **Gene name** | **Unigene ID** | **Unigene ORF (aa)** | **BLASTx best hit** | **Query cover** | **E** | **Ident** | **The Length of "Best hit" (aa)** | **ID** |
| --- | --- | --- | --- | --- | --- | --- | --- | --- | --- |
| 1 | GsinOBP12 | TRINITY_DN40247_c0_g1 | 255 | general odorant-binding protein 71 isoform X1 [Cryptotermes secundus] | 92% | 2.00E-39 | 33.94% | 279 | XP_023702229.1 |
| 2 | GsinOBP8 | TRINITY_DN11893_c0_g4 | 160 | general odorant-binding protein 57c isoform X2 [Zootermopsis nevadensis] | 90% | 9.00E-49 | 54.30% | 155 | XP_021924930.1 |
| 3 | GsinOBP14b | TRINITY_DN38480_c0_g1 | 141 | general odorant-binding protein 56a-like [Cryptotermes secundus] | 95% | 6.00E-18 | 33.81% | 152 | XP_023719059.1 |
| 4 | GsinOBP1 | TRINITY_DN2807_c0_g4 | 147 | odorant-binding protein 8 [Sogatella furcifera] | 92% | 2.00E-17 | 33.09% | 143 | AHB59654.1 |
| 5 | GsinOBP30a | TRINITY_DN39850_c0_g1 | 154 | general odorant-binding protein 69a-like [Zootermopsis nevadensis] | 98% | 3.00E-53 | 54.97% | 150 | XP_021937237.1 |
| 6 | GsinOBP9 | TRINITY_DN25343_c1_g1 | 250 | general odorant-binding protein 83a-like [Athalia rosae] | 82% | 2.00E-28 | 47.37% | 136 | XP_012265247.2 |
| 7 | GsinOBP14 | TRINITY_DN3476_c0_g1 | 161 | general odorant-binding protein 84a-like [Zootermopsis nevadensis] | 68% | 1.00E-31 | 46.36% | 143 | XP_021916985.1 |
| 8 | GsinOBP89 | TRINITY_DN6898_c0_g2 | 247 | odorant-binding protein 15 [Chrysopa pallens] | 90% | 5.00E-62 | 45.54% | 256 | AKM52555.1 |
| 9 | GsinOBP56 | TRINITY_DN40069_c0_g1 | 134 | general odorant-binding protein 56a-like [Schistocerca serialis cubense] | 94% | 3.00E-11 | 29.13% | 146 | XP_049955963.1 |
| 10 | GsinOBP30b | TRINITY_DN8046_c0_g1 | 152 | odorant binding protein 7 [Coptotermes formosanus] | 94% | 9.00E-48 | 53.79% | 151 | WCP86623.1 |
| 11 | GsinOBP24 | TRINITY_DN5322_c0_g1 | 192 | odorant binding protein 8 [Subpsaltria yangi] | 91% | 6.00E-15 | 29.63% | 240 | AXY87867.1 |

**Table S8. List of CSP genes in *G. sinensis***

| **Number** | **Gene name** | **Unigene ID** | **Unigene ORF (aa)** | **BLASTx best hit** | **Query cover** | **E** | **Ident** | **The Length of "Best hit" (aa)** | **ID** |
| --- | --- | --- | --- | --- | --- | --- | --- | --- | --- |
| 1 | GsinCSP16 | TRINITY_DN39176_c0_g1 | 122 | chemosensory protein 6 [Matsumurasca onukii] | 97% | 1.00E-29 | 42.86% | 132 | AWC68037.1 |
| 2 | GsinCSP8 | TRINITY_DN168_c6_g1 | 140 | chemosensory binding protein 1 [Ceracris nigricornis] | 70% | 2.00E-45 | 68.69% | 148 | QHR83077.1 |
| 3 | GsinCSP6 | TRINITY_DN2310_c0_g1 | 124 | chemosensory protein 2 [Dendroctonus armandi] | 95% | 2.00E-39 | 48.76% | 121 | AXF53965.1 |

**Table S9. List of SNMP genes in *G. sinensis***

| **Number** | **Gene name** | **Unigene ID** | **Unigene ORF (aa)** | **BLASTx best hit** | **Query cover** | **E** | **Ident** | **The Length of "Best hit" (aa)** | **ID** |
| --- | --- | --- | --- | --- | --- | --- | --- | --- | --- |
| 1 | GsinSNMP1b | TRINITY_DN36413_c0_g1 | 229 | sensory neuron membrane protein 1 [Odontotermes formosanus] | 100% | 8.00E-79 | 52.84% | 496 | WNX29033.1 |
| 2 | GsinSNMP1a | TRINITY_DN36413_c0_g2 | 129 | sensory neuron membrane protein 1-like [Halyomorpha halys] | 76% | 1.00E-32 | 57.58% | 454 | XP_024216297.1 |
| 3 | GsinSNMP2 | TRINITY_DN5575_c1_g1 | 561 | Sensory neuron membrane protein 2 [Blattella germanica] | 88% | 7.00E-152 | 44.73% | 526 | PSN50836.1 |

**Table S10. List of VSX genes in *G. sinensis***

| **Number** | **Gene name** | **Unigene ID** | **Unigene ORF (aa)** | **BLASTx best hit** | **Query cover** | **E** | **Ident** | **The Length of "Best hit" (aa)** | **ID** |
| --- | --- | --- | --- | --- | --- | --- | --- | --- | --- |
| 1 | GsinVSX2 | TRINITY_DN2508_c1_g1 | 232 | Visual system homeobox 2 [Zootermopsis nevadensis] | 100.00% | 6E-100 | 73.19% | 245 | KDR24461.1 |

**Table S11. List of opsin genes in *G. sinensis***

| **Number** | **Gene name** | **Unigene ID** | **Unigene ORF (aa)** | **BLASTx best hit** | **Query cover** | **E** | **Ident** | **The Length of "Best hit" (aa)** | **ID** |
| --- | --- | --- | --- | --- | --- | --- | --- | --- | --- |
| 1 | Galloisiana sinensis LW | TRINITY_DN11866_c0_g2 | 375 | putative green opsin 1 [Periplaneta americana] | 100.00% | 0.00E+00 | 85.90% | 376 | AKZ20952.1 |
| 2 | Galloisiana sinensis UV | TRINITY_DN17403_c0_g1 | 63 | opsin, ultraviolet-sensitive-like [Homalodisca vitripennis] | 95.00% | 6.00E-22 | 78.33% | 405 | XP_046667175.1 |
| 3 | Galloisiana sinensis Rh5 | TRINITY_DN11008_c0_g1 | 351 | melanopsin-like [Schistocerca gregaria] | 88.00% | 8E-72 | 39.81% | 355 | XP_049846576.1 |

**Table S12. List of PDE6D genes in *G. sinensis***

| **Number** | **Gene name** | **Unigene ID** | **Unigene ORF (aa)** | **BLASTx best hit** | **Query cover** | **E** | **Ident** | **The Length of "Best hit" (aa)** | **ID** |
| --- | --- | --- | --- | --- | --- | --- | --- | --- | --- |
| 1 | GsinPDE6D | TRINITY_DN2960_c0_g2 | 147 | retinal rod rhodopsin-sensitive cGMP 3',5'-cyclic phosphodiesterase subunit delta isoform X1 [Cryptotermes secundus] | 100.00% | 3.00E-99 | 92.52% | 147 | XP_023708891.1 |

**Table S13. List of RBP genes in *G. sinensis***

| **Number** | **Gene name** | **Unigene ID** | **Unigene ORF (aa)** | **BLASTx best hit** | **Query cover** | **E** | **Ident** | **The Length of "Best hit" (aa)** | **ID** |
| --- | --- | --- | --- | --- | --- | --- | --- | --- | --- |
| 1 | GsinRBP | TRINITY_DN9706_c0_g2 | 317 | Retinol-binding protein pinta [Blattella germanica] | 97.00% | 6.00E-132 | 61.09% | 289 | PSN50561.1 |

**Table S14. List of Tret genes in *G. sinensis***

| **Number** | **Gene name** | **Unigene ID** | **Unigene ORF (aa)** | **BLASTx best hit** | **Query cover** | **E** | **Ident** | **The Length of "Best hit" (aa)** | **ID** |
| --- | --- | --- | --- | --- | --- | --- | --- | --- | --- |
| 1 | GsinTret1 TRINITY_DN8201_c1_g1 | TRINITY_DN8201_c1_g1 | 508 | facilitated trehalose transporter Tret1-2 homolog [Cryptotermes secundus] | 94% | 5.00E-155 | 47.29% | 492 | XP_023704097.1 |
| 2 | GsinTret1 TRINITY_DN11828_c0_g1 | TRINITY_DN11828_c0_g1 | 462 | facilitated trehalose transporter Tret1 [Cryptotermes secundus] | 87% | 3.00E-87 | 39.37% | 469 | XP_023714937.1 |
| 3 | GsinTret1 TRINITY_DN16695_c0_g1 | TRINITY_DN16695_c0_g1 | 520 | facilitated trehalose transporter Tret1-2 homolog isoform X2 [Cryptotermes secundus] | 100% | 0 | 76.05% | 526 | XP_023702264.1 |
| 4 | GsinTret1 TRINITY_DN3652_c0_g3 | TRINITY_DN3652_c0_g3 | 530 | facilitated trehalose transporter Tret1-2 homolog isoform X1 [Cryptotermes secundus] | 94% | 0 | 63.06% | 553 | XP_023718925.1 |
| 5 | GsinTret1 TRINITY_DN2446_c2_g1 | TRINITY_DN2446_c2_g1 | 535 | facilitated trehalose transporter Tret1-2 homolog isoform X2 [Zootermopsis nevadensis] | 100% | 0 | 65.61% | 529 | XP_021926811.1 |
| 6 | GsinTret1 TRINITY_DN8201_c1_g2 | TRINITY_DN8201_c1_g2 | 505 | facilitated trehalose transporter Tret1-2 homolog [Cryptotermes secundus] | 95% | 5.00E-152 | 47.22% | 492 | XP_023704097.1 |
| 7 | GsinTret1 TRINITY_DN95_c3_g1 | TRINITY_DN95_c3_g1 | 516 | facilitated trehalose transporter Tret1-2 homolog [Zootermopsis nevadensis] | 94% | 8.00E-141 | 45.83% | 495 | XP_021938022.1 |
| 8 | GsinTret1 TRINITY_DN3053_c1_g1 | TRINITY_DN3053_c1_g1 | 350 | Facilitated trehalose transporter Tret1 [Blattella germanica] | 93% | 9.00E-120 | 51.53% | 420 | PSN31408.1 |
| 9 | GsinTret1 TRINITY_DN1262_c1_g1 | TRINITY_DN1262_c1_g1 | 500 | facilitated trehalose transporter Tret1-like [Zootermopsis nevadensis] | 95% | 6.00E-170 | 51.86% | 508 | XP_021940558.1 |
| 10 | GsinTret1 TRINITY_DN13995_c0_g2 | TRINITY_DN13995_c0_g2 | 415 | facilitated trehalose transporter Tret1-2 homolog [Zootermopsis nevadensis] | 98% | 2.00E-135 | 50.36% | 495 | XP_021938022.1 |
| 11 | GsinTret1 TRINITY_DN1795_c1_g1 | TRINITY_DN1795_c1_g1 | 510 | facilitated trehalose transporter Tret1-2 homolog [Bradysia coprophila] | 95% | 0 | 51.53% | 532 | XP_037043245.1 |
| 12 | GsinTret1 TRINITY_DN5760_c0_g1 | TRINITY_DN5760_c0_g1 | 559 | facilitated trehalose transporter Tret1-2 homolog isoform X2 [Schistocerca gregaria] | 89% | 0 | 56.97% | 522 | XP_049845304.1 |
| 13 | GsinTret1 TRINITY_DN1858_c0_g2 | TRINITY_DN1858_c0_g2 | 547 | Facilitated trehalose transporter Tret1 [Blattella germanica] | 95% | 2.00E-137 | 42.56% | 469 | PSN35073.1 |
| 14 | GsinTret1 TRINITY_DN9083_c0_g1 | TRINITY_DN9083_c0_g1 | 485 | facilitated trehalose transporter Tret1-like [Zootermopsis nevadensis] | 97% | 2.00E-98 | 36.42% | 571 | XP_021936744.1 |
| 15 | GsinTret1 TRINITY_DN1173_c0_g1 | TRINITY_DN1173_c0_g1 | 524 | Facilitated trehalose transporter Tret1-2 [Blattella germanica] | 84% | 2.00E-170 | 56.40% | 443 | PSN45090.1 |
| 16 | GsinTret1 TRINITY_DN1173_c0_g3 | TRINITY_DN1173_c0_g3 | 524 | Facilitated trehalose transporter Tret1-2 [Blattella germanica] | 85% | 3.00E-161 | 53.79% | 443 | PSN45090.1 |
| 17 | GsinTret1 TRINITY_DN6516_c0_g1 | TRINITY_DN6516_c0_g1 | 516 | facilitated trehalose transporter Tret1-2 homolog isoform X1 [Zootermopsis nevadensis] | 98% | 0 | 63.81% | 515 | XP_021923673.1 |
| 18 | GsinTret1 TRINITY_DN16682_c0_g1 | TRINITY_DN16682_c0_g1 | 367 | facilitated trehalose transporter Tret1-2 homolog isoform X2 [Cryptotermes secundus] | 86% | 9.00E-80 | 43.48% | 608 | XP_023724431.1 |
| 19 | GsinTret1 TRINITY_DN1395_c0_g3 | TRINITY_DN1395_c0_g3 | 504 | facilitated trehalose transporter Tret1-like [Cryptotermes secundus] | 100% | 0 | 84.39% | 501 | XP_033609139.1 |
| 20 | GsinTret1 TRINITY_DN7820_c0_g1 | TRINITY_DN7820_c0_g1 | 430 | facilitated trehalose transporter Tret1 [Cryptotermes secundus] | 99% | 9.00E-157 | 54.76% | 459 | XP_023719427.1 |
| 21 | GsinTret1 TRINITY_DN6568_c0_g1 | TRINITY_DN6568_c0_g1 | 614 | Facilitated trehalose transporter Tret1 [Blattella germanica] | 96% | 0 | 63.67% | 590 | PSN42188.1 |
| 22 | GsinTret1 TRINITY_DN5083_c0_g1 | TRINITY_DN5083_c0_g1 | 566 | facilitated trehalose transporter Tret1 [Cryptotermes secundus] | 97% | 0 | 55.08% | 527 | XP_023714308.1 |
| 23 | GsinTret1 TRINITY_DN16205_c2_g1 | TRINITY_DN16205_c2_g1 | 91 | facilitated trehalose transporter Tret1-like isoform X2 [Zootermopsis nevadensis] | 100% | 9.00E-38 | 79.12% | 489 | XP_021922628.1 |
| 24 | GsinTret1 TRINITY_DN2213_c1_g1 | TRINITY_DN2213_c1_g1 | 478 | Facilitated trehalose transporter Tret1 [Blattella germanica] | 97% | 0 | 60.70% | 492 | PSN47391.1 |
| 25 | GsinTret1 TRINITY_DN6484_c5_g1 | TRINITY_DN6484_c5_g1 | 544 | facilitated trehalose transporter Tret1-like isoform X1 [Zootermopsis nevadensis] | 97% | 0 | 65.02% | 632 | XP_021920859.1 |
| 26 | GsinTret1 TRINITY_DN758_c2_g1 | TRINITY_DN758_c2_g1 | 516 | facilitated trehalose transporter Tret1 isoform X1 [Cryptotermes secundus] | 97% | 3.00E-146 | 45.63% | 490 | XP_023719424.1 |

**Table S15. List of Trp genes in *G. sinensis***

| **Number** | **Gene name** | **Unigene ID** | **Unigene ORF (aa)** | **BLASTx best hit** | **Query cover** | **E** | **Ident** | **The Length of "Best hit" (aa)** | **ID** |
| --- | --- | --- | --- | --- | --- | --- | --- | --- | --- |
| 1 | Gsin TRPV TRINITY_DN15039_c0_g1 | TRINITY_DN15039_c0_g1 | 910 | transient receptor potential channel pyrexia isoform X1 [Cryptotermes secundus] | 96% | 0 | 64.96% | 949 | XP_023718298.1 |
| 2 | Gsin TRPC TRINITY_DN14244_c0_g1 | TRINITY_DN14244_c0_g1 | 256 | transient receptor potential cation channel protein painless isoform X2 [Cryptotermes secundus] | 96% | 5.00E-32 | 34.46% | 961 | XP_023714372.1 |
| 3 | Gsin TRPV TRINITY_DN32144_c0_g1 | TRINITY_DN32144_c0_g1 | 835 | transient receptor potential channel pyrexia [Cryptotermes secundus] | 98% | 0 | 48.91% | 894 | XP_023716721.1 |
| 4 | Gsin TRPV TRINITY_DN29949_c1_g1 | TRINITY_DN29949_c1_g1 | 256 | transient receptor potential cation channel protein painless-like [Galleria mellonella] | 98% | 4.00E-12 | 26.94% | 953 | XP_031763294.2 |
| 5 | Gsin TRPV TRINITY_DN21455_c0_g2 | TRINITY_DN21455_c0_g2 | 229 | transient receptor potential cation channel protein painless-like [Zootermopsis nevadensis] | 97% | 4.00E-33 | 36.40% | 998 | XP_021924797.1 |
| 6 | Gsin TRPV TRINITY_DN7033_c0_g1 | TRINITY_DN7033_c0_g1 | 705 | transient receptor potential cation channel protein painless-like [Zootermopsis nevadensis] | 99% | 0 | 47.08% | 998 | XP_021924797.1 |
| 7 | Gsin TRPC TRINITY_DN19526_c0_g1 | TRINITY_DN19526_c0_g1 | 149 | Transient receptor potential cation channel subfamily A member 1 [Cryptotermes secundus] | 100% | 3.00E-83 | 83.22% | 552 | PNF38880.1 |
| 8 | Gsin TRPC TRINITY_DN38426_c0_g1 | TRINITY_DN38426_c0_g1 | 174 | Transient receptor potential cation channel protein painless [Gryllus bimaculatus] | 98% | 5.00E-15 | 28.91% | 1046 | GLG98515.1 |
| 9 | Gsin TRPC TRINITY_DN2470_c4_g2 | TRINITY_DN2470_c4_g2 | 848 | Transient receptor potential cation channel protein painless [Gryllus bimaculatus] | 94% | 9.00E-122 | 32.67% | 1046 | GLG98515.1 |
| 10 | Gsin TRPV TRINITY_DN12933_c0_g1 | TRINITY_DN12933_c0_g1 | 717 | transient receptor potential channel pyrexia-like [Neodiprion virginianus] | 99% | 5.00E-171 | 40.00% | 1128 | XP_046606728.1 |
| 11 | Gsin TRPC TRINITY_DN7033_c0_g3 | TRINITY_DN7033_c0_g3 | 286 | transient receptor potential cation channel protein painless-like [Zootermopsis nevadensis] | 98% | 1.00E-58 | 39.37% | 998 | XP_021924797.1 |
| 12 | Gsin TRPTγ TRINITY_DN27499_c0_g1 | TRINITY_DN27499_c0_g1 | 115 | transient receptor potential-gamma protein [Bacillus rossius redtenbacheri] | 97% | 2.00E-20 | 50.78% | 1153 | XP_063232007.1 |
| 13 | Gsin TRPC TRINITY_DN4038_c0_g2 | TRINITY_DN4038_c0_g2 | 690 | putative TRP channel protein [Periplaneta americana] | 86% | 0 | 68.70% | 1134 | AGG86916.1 |
| 14 | Gsin TRPC TRINITY_DN21455_c0_g1 | TRINITY_DN21455_c0_g1 | 55 | Transient receptor potential cation channel protein painless [Frankliniella fusca] | 100% | 9.00E-12 | 56.45% | 1089 | KAK3932439.1 |
| 15 | Gsin TRPC TRINITY_DN4469_c1_g2 | TRINITY_DN4469_c1_g2 | 1326 | transient receptor potential cation channel trpm isoform X8 [Bacillus rossius redtenbacheri] | 100% | 0 | 77.17% | 1690 | XP_063221528.1 |
| 16 | Gsin TRPV TRINITY_DN39524_c0_g1 | TRINITY_DN39524_c0_g1 | 149 | transient receptor potential Pain [Bemisia tabaci] | 97% | 2.00E-30 | 47.95% | 965 | WMY99263.1 |
| 17 | Gsin TRPC TRINITY_DN26176_c0_g1 | TRINITY_DN26176_c0_g1 | 163 | transient receptor potential protein-like [Halyomorpha halys] | 88% | 2.00E-79 | 82.07% | 299 | XP_014293074.1 |
| 18 | Gsin TRPC TRINITY_DN4677_c0_g1 | TRINITY_DN4677_c0_g1 | 989 | transient receptor potential channel pyrexia [Bacillus rossius redtenbacheri] | 97% | 0 | 58.03% | 974 | XP_063225666.1 |
| 19 | Gsin TRPC TRINITY_DN88_c20_g1 | TRINITY_DN88_c20_g1 | 995 | transient receptor potential cation channel protein painless-like [Zootermopsis nevadensis] | 100% | 0 | 44.71% | 998 | XP_021924797.1 |

**Table S16. List of HSP genes in *G. sinensis***

| **Number** | **Gene name** | **Unigene ID** | **Unigene ORF (aa)** | **BLASTx best hit** | **Query cover** | **E** | **Ident** | **The Length of "Best hit" (aa)** | **ID** |
| --- | --- | --- | --- | --- | --- | --- | --- | --- | --- |
| 1 | GsinHSP17.3 | TRINITY_DN18717_c0_g1 | 150 | 18.2 kDa class I heat shock protein-like [Pistacia vera] | 100% | 1.00E-90 | 85.16% | 154 | XP_031286243.1 |
| 2 | GsinHSP56.1 | TRINITY_DN3551_c0_g1 | 505 | heat shock 70 kDa protein 14 [Cryptotermes secundus] | 98% | 1.00E-165 | 48.71% | 502 | XP_023715279.1 |
| 3 | GsinHSP75.2 | TRINITY_DN21497_c0_g1 | 689 | Heat shock 70 kDa protein cognate 5 [Blattella germanica] | 95% | 0 | 87.58% | 682 | PSN32841.1 |
| 4 | GsinHSP18.6 | TRINITY_DN2717_c0_g1 | 168 | heat shock protein 90 [Neocloeon triangulifer] | 100% | 4.00E-106 | 91.07% | 224 | AEE01388.1 |
| 5 | GsinHSP91.8 | TRINITY_DN4027_c1_g1 | 813 | 97 kDa heat shock protein [Cryptotermes secundus] | 100% | 0 | 76.29% | 828 | XP_023714084.1 |
| 6 | GsinHSP15.8 | TRINITY_DN5348_c1_g1 | 724 | heat shock protein 83 [Cryptotermes secundus] | 100% | 0 | 89.96% | 727 | XP_023720867.1 |
| 7 | GsinHSP83.2 | TRINITY_DN27067_c0_g2 | 55 | 70 kDa heat shock protein [Cornitermes cumulans] | 100% | 9.00E-31 | 100.00% | 87 | AZA04938.1 |
| 8 | GsinHSP6.2 | TRINITY_DN19718_c0_g1 | 89 | Hsp70 family protein [Staphylococcus aureus] | 100% | 7.00E-54 | 100.00% | 121 | MBO8666295.1 |
| 9 | GsinHSP10.4 | TRINITY_DN27067_c0_g1 | 100 | Hsp70 family protein [Pseudomonas syringae] | 100% | 5.00E-65 | 100.00% | 169 | WP_158103049.1 |
| 10 | GsinHSP11.7 | TRINITY_DN17860_c0_g1 | 87 | heat shock protein HSP 90-alpha-like [Microtus fortis] | 100% | 7.00E-56 | 100.00% | 124 | XP_050007478.1 |
| 11 | GsinHSP9.7 | TRINITY_DN3640_c1_g1 | 167 | LOW QUALITY PROTEIN: 15.7 kDa heat shock protein, peroxisomal-like [Schistocerca piceifrons] | 100% | 3.00E-31 | 41.14% | 172 | XP_047120797.1 |
| 12 | GsinHSP19.1 | TRINITY_DN5212_c0_g1 | 706 | heat shock protein 75 kDa, mitochondrial [Cryptotermes secundus] | 100% | 0 | 75.81% | 704 | XP_023725398.1 |
| 13 | GsinHSP79.7 | TRINITY_DN4309_c0_g1 | 638 | heat shock protein 70c [Paratlanticus ussuriensis] | 99% | 0 | 79.31% | 640 | AFP54305.1 |
| 14 | GsinHSP70.2 | TRINITY_DN42074_c0_g1 | 134 | Alpha crystallin/Hsp20 domain - like 10 [Theobroma cacao] | 68% | 1.00E-23 | 56.52% | 233 | WRX33569.1 |
| 15 | GsinHSP17.7 | TRINITY_DN38796_c0_g1 | 154 | small heat-shock protein [Pseudotsuga menziesii] | 90% | 6.00E-86 | 88.81% | 161 | CAC81964.1 |
| 16 | GsinHSP74.2 | TRINITY_DN2645_c0_g2 | 670 | heat shock 70 kDa protein cognate 3 [Zootermopsis nevadensis] | 98% | 0 | 94.82% | 655 | XP_021933543.1 |
| 17 | GsinHSP17.3b | TRINITY_DN13016_c0_g1 | 156 | 17.3 kDa class II heat shock protein-like [Quillaja saponaria] | 100% | 4.00E-95 | 87.26% | 157 | KAJ7966392.1 |
| 18 | GsinHSP17.5 | TRINITY_DN40679_c0_g1 | 150 | 18.2 kDa class I heat shock protein-like [Pistacia vera] | 100% | 1.00E-89 | 84.52% | 154 | XP_031286243.1 |
| 19 | GsinHSP61.4 | TRINITY_DN4850_c0_g6 | 575 | heat shock protein 60A-like [Bacillus rossius redtenbacheri] | 96% | 0 | 84.63% | 572 | XP_063223917.1 |
| 20 | GsinHSP6.7 | TRINITY_DN38484_c0_g1 | 63 | heat shock protein 70 [Felis catus] | 74% | 3.00E-20 | 95.74% | 62 | CCO02800.1 |

**Table S17. List of DnaJ genes in *G. sinensis***

| **Number** | **Gene name** | **Unigene ID** | **Unigene ORF (aa)** | **BLASTx best hit** | **Query cover** | **E** | **Ident** | **The Length of "Best hit" (aa)** | **ID** |
| --- | --- | --- | --- | --- | --- | --- | --- | --- | --- |
| 1 | GsinDNAJA2 | TRINITY_DN1016_c0_g1 | 412 | dnaJ homolog subfamily A member 2-like isoform X2 [Zootermopsis nevadensis] | 100% | 0 | 87.86% | 444 | XP_021928687.1 |
| 2 | GsinDNAJC17 | TRINITY_DN34280_c0_g2 | 223 | dnaJ homolog subfamily C member 17 isoform X2 [Thrips palmi] | 100% | 5.00E-122 | 54.70% | 350 | XP_034252926.1 |
| 3 | GsinDNAJC1 | TRINITY_DN6664_c0_g1 | 232 | dnaJ homolog subfamily C member 1 [Zootermopsis nevadensis] | 97% | 0 | 65.93% | 464 | XP_021940138.1 |
| 4 | GsinDNAJC30 | TRINITY_DN38272_c0_g1 | 257 | dnaJ homolog subfamily C member 30, mitochondrial [Schistocerca serialis cubense] | 85% | 6.00E-65 | 54.15% | 221 | XP_049941324.1 |
| 5 | GsinDNAJC16a | TRINITY_DN33056_c0_g5 | 368 | dnaJ homolog subfamily C member 16 [Zootermopsis nevadensis] | 100% | 1.00E-15 | 74.47% | 818 | XP_021921742.1 |
| 6 | GsinDNAJC3 | TRINITY_DN7803_c0_g1 | 295 | dnaJ homolog subfamily C member 3 [Zootermopsis nevadensis] | 100% | 0 | 75.26% | 488 | XP_021934310.1 |
| 7 | GsinDNAJC28 | TRINITY_DN25128_c1_g1 | 139 | dnaJ homolog subfamily C member 28 isoform X1 [Zootermopsis nevadensis] | 92% | 2.00E-136 | 56.35% | 442 | XP_021932984.1 |
| 8 | GsinDNAJC22 | TRINITY_DN2685_c0_g1 | 351 | dnaJ homolog subfamily C member 22 [Cryptotermes secundus] | 98% | 0 | 72.49% | 355 | XP_023726511.1 |
| 9 | GsinDNAJC24 | TRINITY_DN34411_c0_g1 | 928 | DnaJ subfamily C member 24 [Cryptotermes secundus] | 100% | 2.00E-38 | 49.64% | 165 | PNF22226.1 |
| 10 | GsinDNAJshv | TRINITY_DN20234_c1_g2 | 489 | dnaJ homolog shv [Schistocerca piceifrons] | 99% | 0 | 80.00% | 376 | XP_047107020.1 |
| 11 | GsinDNAJ1a | TRINITY_DN17389_c0_g1 | 449 | DnaJ protein homolog 1 [Gryllus bimaculatus] | 100% | 0 | 86.72% | 372 | GLH00743.1 |
| 12 | GsinDNAJB6 | TRINITY_DN438_c0_g1 | 264 | dnaJ homolog subfamily B member 6 isoform X1 [Cryptotermes secundus] | 100% | 1.00E-108 | 63.12% | 285 | XP_023712920.1 |
| 13 | GsinDNAJC5 | TRINITY_DN91_c0_g1 | 615 | dnaJ homolog subfamily C member 5 isoform X2 [Zootermopsis nevadensis] | 100% | 5.00E-122 | 75.49% | 242 | XP_021927420.1 |
| 14 | GsinDNAJB12 | TRINITY_DN8099_c0_g1 | 495 | dnaJ homolog subfamily B member 12 isoform X1 [Cryptotermes secundus] | 100% | 0 | 75.85% | 378 | XP_023712622.1 |
| 15 | GsinDNAJC14 | TRINITY_DN5705_c0_g1 | 564 | DnaJ-like protein subfamily C member 14 [Zootermopsis nevadensis] | 94% | 0 | 54.14% | 882 | KDR16318.1 |
| 16 | GsinDNAJB9 | TRINITY_DN33682_c1_g1 | 964 | dnaJ homolog subfamily B member 9 [Cryptotermes secundus] | 88% | 3.00E-85 | 67.68% | 210 | XP_023708596.1 |
| 17 | GsinDNAJC13a | TRINITY_DN18549_c1_g4 | 47 | dnaJ homolog subfamily C member 13 [Bacillus rossius redtenbacheri] | 99% | 0 | 73.77% | 2235 | XP_063220868.1 |
| 18 | GsinDNAJC13b | TRINITY_DN18549_c1_g2 | 235 | DnaJ sub C member 13 [Periplaneta americana] | 100% | 9.00E-149 | 81.82% | 1544 | KAJ4448169.1 |
| 19 | GsinDNAJC10 | TRINITY_DN3863_c0_g1 | 767 | dnaJ homolog subfamily C member 10-like [Zootermopsis nevadensis] | 99% | 0 | 63.21% | 782 | XP_021926361.1 |
| 20 | GsinDNAJC9 | TRINITY_DN20249_c0_g2 | 392 | DnaJ subfamily C member 9 [Blattella germanica] | 97% | 7.00E-113 | 69.38% | 258 | PSN33465.1 |
| 21 | GsinDNAJC11 | TRINITY_DN5839_c0_g1 | 298 | dnaJ homolog subfamily C member 11 isoform X1 [Zootermopsis nevadensis] | 100% | 0 | 74.09% | 575 | XP_021920736.1 |
| 22 | GsinDNAJ1b | TRINITY_DN33056_c0_g3 | 250 | DnaJ protein homolog 1 [Gryllus bimaculatus] | 100% | 1.00E-165 | 75.92% | 808 | GLG99658.1 |
| 23 | GsinDNAJC13 | TRINITY_DN25467_c0_g2 | 356 | dnaJ homolog subfamily C member 13 isoform X1 [Cryptotermes secundus] | 100% | 1.00E-135 | 87.93% | 1966 | XP_023705144.1 |
| 24 | GsinDNAJC16b | TRINITY_DN33056_c0_g2 | 286 | dnaJ homolog subfamily C member 16 isoform X2 [Cryptotermes secundus] | 100% | 4.00E-149 | 70.86% | 818 | XP_023727201.1 |
| 25 | GsinDNAJC7 | TRINITY_DN1090_c0_g1 | 342 | dnaJ homolog subfamily C member 7 [Cryptotermes secundus] | 97% | 0 | 78.05% | 496 | XP_023718809.1 |
| 26 | GsinDNAJC2 | TRINITY_DN234_c0_g2 | 354 | dnaJ homolog subfamily C member 2 isoform X2 [Zootermopsis nevadensis] | 97% | 0 | 66.06% | 609 | XP_021939402.1 |

**Table S18. List of Wnt genes in *G. sinensis***

| **Number** | **Gene name** | **Unigene ID** | **Unigene ORF (aa)** | **BLASTx best hit** | **Query cover** | **E** | **Ident** | **The Length of "Best hit" (aa)** | **ID** |
| --- | --- | --- | --- | --- | --- | --- | --- | --- | --- |
| 1 | GsinWnt7b | TRINITY_DN27608_c0_g1 | 370 | protein Wnt-7b [Cryptotermes secundus] | 97% | 0.00E+00 | 82.87% | 371 | XP_023715845.1 |
| 2 | GsinWnt16 | TRINITY_DN1853_c0_g1 | 390 | protein Wnt-16-like [Homalodisca vitripennis] | 93% | 0 | 79.89% | 385 | XP_046684210.1 |
| 3 | GsinWnt6 | TRINITY_DN1372_c0_g1 | 354 | protein Wnt-6 [Cryptotermes secundus] | 100% | 0.00E+00 | 83.33% | 359 | XP_023701471.1 |
